# Interaction Mechanisms and Predictions of the Biofouling of Polymer Films: A Combined Atomic Force Microscopy and Quartz Crystal Microbalance with Dissipation Monitoring Study

**DOI:** 10.1021/acs.langmuir.3c00587

**Published:** 2023-04-27

**Authors:** Asma Eskhan, Neveen AlQasas, Daniel Johnson

**Affiliations:** † NYUAD Water Research Center, New York University Abu Dhabi (NYUAD), 129188 Abu Dhabi, UAE; ‡ Division of Engineering, New York University Abu Dhabi, 129188 Abu Dhabi, UAE

## Abstract

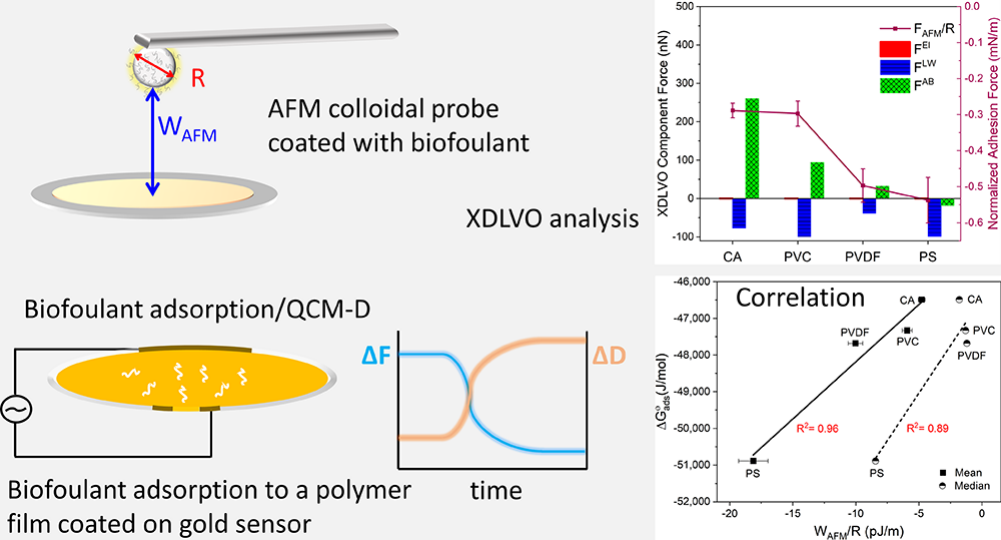

Biofouling of polymeric membranes is a severe problem in water desalination and treatment applications. A fundamental understanding of biofouling mechanisms is necessary to control biofouling and develop more efficient mitigation strategies. To shed light on the type of forces that govern the interactions between biofoulants and membranes, biofoulant-coated colloidal AFM probes were employed to investigate the biofouling mechanisms of two model biofoulants, BSA and HA, toward an array of polymer films commonly used in membrane synthesis, which included CA, PVC, PVDF, and PS. These experiments were combined with quartz crystal microbalance with dissipation monitoring (QCM-D) measurements. The Derjaguin, Landau, Verwey, and Overbeek (DLVO) and the extended-DLVO (XDLVO) theoretical models were applied to decouple the overall adhesion interactions between the biofoulants and the polymer films into their component interactions, i.e., electrostatic (El), Lifshitz–van der Waals (LW), and Lewis acid–base (AB) interactions. The XDLVO model was found to predict better the AFM colloidal probe adhesion data and the QCM-D adsorption behavior of BSA onto the polymer films than the DLVO model. The ranking of the polymer films’ adhesion strengths and adsorption quantities was inversely proportional to their γ^–^ values. Higher normalized adhesion forces were quantified for the BSA-coated colloidal probes with the polymer films than the HA-coated colloidal probes. Similarly, in QCM-D measurements, BSA was found to cause larger adsorption mass shifts, faster adsorption rates, and more condensed fouling layers than HA. A linear correlation (*R*^2^ = 0.96) was obtained between the adsorption standard free energy changes (Δ*G*_ads_^°^) estimated for BSA from the equilibrium QCM-D adsorption experiments and the AFM normalized adhesion energies (*W*_AFM_/*R*) estimated for BSA from the AFM colloidal probe measurements. Eventually, an indirect approach was presented to calculate the surface energy components of biofoulants characterized by high porosities from Hansen dissolution tests to perform the DLVO/XDLVO analyses.

## Introduction

Biofouling is an intractable problem in water-membrane systems, which ultimately results in a loss of membrane performance. It can be described as the undesired deposition and growth of biofilms on surfaces.^1^ This consequently leads to a drastic decline in permeate flux and salt rejection, making periodic replacement of membranes necessary. The formation of biofilms on surfaces usually takes place in four consecutive steps: (i) adsorption of nutrients and organic matter in the feed solution onto the membrane surface, forming the so-called conditioning film, which is sometimes classified as organic fouling; (ii) initial reversible/irreversible adhesion of microorganisms to the conditioned membrane surface; (iii) maturation of the biofilm by multiplication and secretion of a matrix of biomacromolecules; and (iv) detachment of the biofilm.^2^ The conditioning layer can significantly impact the initial bacterial adhesion, either positively or negatively, due to the modification of the physicochemical characteristics of the membrane surface, such as charge, hydrophobicity, and roughness.^3−5^ The initial adhesion of microorganism cells to a membrane surface or a conditioned membrane surface is believed to be a fundamental step in biofouling. However, the mechanisms that govern the interactions involved in the first two steps of biofilm formation are not fully understood. Although several antimicrobial membrane modification strategies have been developed to control biofouling, deposition of conditioning films on membrane surfaces that have been modified can alter the surface chemistry of the modified membrane surface, resulting in a loss or deactivation of its anti-bacterial resistance. Therefore, it is critical to understand the interactions at a fundamental level between biofoulants that are present in the feed solution and the membrane surface. Atomic force microscopy (AFM) and quartz crystal microbalance with dissipation monitoring (QCM-D) are two powerful techniques that can be applied to better understand the processes occurring at the membrane surface/interface, such as the biofouling process. Both these techniques are label-free and strongly sensitive to allow quantification and detection of the adhesion of biofoulants to membrane surfaces. (For more details on both techniques, see a review by Bonet et al.).^6^

AFM is a versatile tool for high-resolution characterization of membrane surface properties, including membrane surface roughness, pore size, and pore size distribution.^7^ AFM can also be applied to performing interfacial force measurements in the pico-Newton range providing information on the interactions interplaying between the membrane surface and AFM probes in air and liquid environments. AFM colloidal probes are sometimes favored over standard AFM sharp tips due to their well-known geometry and size, higher sensitivity to weak interactions, and more extensive surface area available for coating or functionalization, thus offering a suitable solution for evaluating the biofouling propensity of surfaces.^8^ The colloidal probe can be functionalized with the biofoulant of interest or with individual living cells and retracted from the membrane surface to quantify the interplaying interactions, which would aid in understanding the mechanisms implicated in the biofouling of membranes.^9−12^ In an alternative approach, the colloidal probe can be coated with a polymeric membrane material, and its adhesion to a biofoulant deposited on a membrane sample (a fouled membrane) can be measured.^13,14^ Likewise, QCM-D can provide real-time measurements of the dynamic adsorption or interaction of biofoulant molecules with the membrane surface.^15−18^ The mass of the adsorbed molecules can be measured as changes in the resonance frequency of a quartz crystal that functions as a piezoelectric transducer. Typically, a 5 MHz quartz sensor is capable of measuring as small as 17.7 ng/cm^2^. Moreover, changes in the energy dissipation of the quartz sensor provide information on the structural and viscoelastic properties of the adsorbed layer.^6^

Shortage of clean water resources is a globally encountered challenge. Thereby, the demand for energy-efficient and sustainable water treatment is continuously growing. Compared to conventional water purification techniques, such as thermal processes, membrane-based processes can offer cost-effective solutions that require comparatively low amounts of energy.^19^ However, the optimization of membrane-based water treatment processes is still limited by the major bottleneck of fouling and scaling problems. During recent years, polymeric membranes have attracted much interest due to their chemical and thermal stabilities, mechanical strength, and flexibility.^20^ Although the permeability and selectivity of the polymeric membranes have been improved by introducing second phases of polymers and nanofillers,^21^ an additional improvement in their anti-biofouling properties is still required to expand their application in water treatment processes further.

To better comprehend the mechanisms underlying the biofouling of polymeric membranes and understand the involved interactions, we used the AFM colloidal probe technique and QCM-D to measure the biofouling potential of two model biofoulants, i.e., bovine serum albumin (BSA) and humic acid (HA), toward a group of polymer films, namely, cellulose acetate (CA), polyvinyl chloride (PVC), polyvinylidene fluoride (PVDF), and polysulfone (PS), commonly used in membrane synthesis. AFM colloidal probes coated with BSA or HA were utilized to measure the adhesion of BSA or HA molecules to the polymer films. In parallel, gold-plated QCM-D sensors were covered with the polymer film of interest and used to measure the BSA and HA adsorption rates and mass quantities. Furthermore, with the help of contact angle and zeta potential measurements, we applied the extended Derjaguin, Landau, Verwey, and Overbeek (XDLVO) analysis that attributes the overall adhesion force to three primary component forces (i.e., electrostatic, Lifshitz–van der Waals, and Lewis acid–base forces), to uncover the predominant forces that are thought to control membrane biofouling.

## Materials and Methods

### Materials

Amine-functionalized silica spheres with a mean diameter of 5.0 μm were purchased from Polysciences Inc., USA. BSA, HA, and albumin–fluorescein isothiocyanate conjugate (FITC-labeled BSA) were obtained from Sigma-Aldrich Inc., USA. Sodium hydroxide (NaOH), hydrochloric acid (HCl), and *N*-methyl-2-pyrrolidone (NMP) were supplied by VWR Chemicals Inc., Germany. The polymers CA, PVC, PVDF, and PS were purchased from Sigma-Aldrich Inc., USA. The UV curable adhesive glue (Loctite 34931) was purchased from Loctite, USA. All the chemicals were used as received without any further purification. The water used in this study was obtained from a Milli-Q IQ 7015 pure and ultrapure water purification system with a produced water resistivity of 18.2 MΩ.cm (termed as DI water in this study).

### Colloidal Probe Functionalization

Amine-functionalized silica spheres were coated with BSA or HA by immersing the spheres in 2000 mg/L of BSA or HA solutions and storing them at 4.0 °C overnight. The BSA solution was prepared by directly dissolving BSA powder in DI water. To dissolve HA in DI water, 0.1 M NaOH was added dropwise to the HA solution until it was completely dissolved. The HA solution was then filtered using a 4.5 μm syringe filter. The pH of the HA solution was adjusted by adding HCl dropwise until the pH meter showed a reading between 7.5 and 8. Next day, the spheres’ suspensions in BSA and HA solutions were centrifuged at 4200*g* for 15 min and twice washed with DI water. A drop of each solution diluted enough to prevent the aggregation of the spheres was placed on a cleaned microscope slide and allowed to dry at room temperature. A sphere on the glass slide was selected and glued to the apex of a tipless AFM cantilever (AIO-TL, BudgetSensors, Bulgaria) using an MSM 400 System micromanipulator (Singer Instruments, UK). The glue was cured under direct sunlight for 1 h. Scanning electron microscopy (SEM, FEI Quanta FEG 450) images at an acceleration voltage of 5.0 k were recorded for each colloidal probe used in each experiment to measure the exact radius of the sphere and to ensure that it is devoid of any defects and protrusions. To ensure that BSA was successfully and uniformly coated on the amine-functionalized silica spheres, fluorescence microscopy imaging was utilized (Leica DMI6000 B). For that, the spheres were coated with FITC-labeled BSA to enable the fluorescent visualization of the coating, following the same procedure used for coating of the spheres with BSA.

### Polymer Film Preparation

Four polymer films, CA, PVC, PVDF, and PS, were prepared by dissolving the corresponding polymer powders in NMP solvent at room temperature for 24 h. The polymer solutions were then poured into a petri dish and heated in an oven (Carbolite Gero Ltd.) at 35 °C for 24–48 h until a dried film was formed. The CA, PVC, PVDF, and PS solutions were prepared in the concentrations of 100, 22.5, 83.3, and 300 g/L, respectively.

### AFM Force Measurements

All force measurements were performed using Bruker Multimode 8 AFM with a NanoScope V controller using a J scanner (Bruker Nano Santa Barbara, Inc., California, USA). Prior to each measurement, the force constant of the cantilever with a sphere attached to its end was calibrated in DI water using the thermal noise method.^22^ The force constant was always found to be comparable to the value provided by the manufacturer (0.2 N/m). The optical lever sensitivity of the cantilever was determined from the slope of the contact region obtained using the colloidal probe cantilever on a sapphire surface. The calibrated cantilever was then engaged in force-volume contact mode with the polymer film surface under DI water. An average of 144 (12 × 12) approach-retraction force cycles were recorded at a cantilever velocity of 26.9 μm/s, scan size of 12 μm, ramp size of 0.5 μm, and maximum loading force of 1 nN. At the end of each experiment, the cantilever was visualized again using the micromanipulator to ensure that the colloidal sphere did not detach during the measurement and was then stored for subsequent SEM imaging. For each polymer film, triplicate measurements on three different locations were carried out and the average values were reported. The measured adhesion forces and energies for each experiment were normalized by the radius of the colloidal probe obtained via SEM imaging.

### Polymer Films Roughness Measurements by AFM

The surface topography and roughness of the prepared polymer films were characterized using a MultiMode 8 AFM with a NanoScope V controller using a J scanner (Bruker Nano Santa Barbara, Inc., California, USA) and which is equipped with ScanAsyst automatic image optimization. A small piece of each film was mounted on a double-sided carbon tape supported on a steel disk. The samples were imaged using ScanAsyst mode (NanoScope 9.7) under ambient air conditions with a silicon tip on a nitride lever (SCANASYST-AIR probe with a spring constant of 0.4 N/m). The *R*_q_ and *R*_a_ roughness values were obtained by analyzing first-order flattened images using NanoScope Analysis software (version 3.0). All values represent averages of triplicate measurements performed on three different locations on the samples’ surfaces (Table S1). All images were acquired on a 10 μm × 10 μm area with a scanning rate of 1.02 Hz and a resolution of 592 × 592 pixels (Figure S2).

### Hansen Dissolution Tests for Model Biofoulants and Polymers

This data was taken from our previous publication.^23^ The well-established Hansen solubility dissolution tests were employed for the four polymer powders (i.e., CA, PVC, PVDF, and PS).^24^ In brief, 20–25 mg of each polymer powder was dissolved in 10 mL of each tested solvent (methanol, ethanol, water, ethyl acetate, acetone, dichloromethane, dimethylformamide, toluene, NMP, dimethyl sulfoxide, ethylene glycol, isopropyl alcohol, acetonitrile, and formamide) and continuously stirred at room temperature overnight. Visual observation of the polymer dissolution in each solvent was recorded, and the solvents were classified as bad or good solvents using the HSPiP software. For BSA, the data was taken from a publication where the ranking of the solvents was carried out based on BSA solubilities measured by UV–vis to get more accurate results.^25^

### Contact Angle Measurements

Contact angles were measured for the biofoulants and the polymer films using the sessile drop technique with a Drop Shape Analyzer (DSA100S, Kruss Scientific). Three probe liquids with known surface tension components (of which two must be polar) were selected to obtain the surface energy components of the biofoulants and the polymer films, using the van Oss–Chaudhury–Good (OCG) approach.^26,27^ This approach determines the three unknowns (the three surface energy components of the surface) by obtaining a system of three equations using at least three liquids with known surface tension components’ values). According to van Oss, it is a prerequisite that the surface tension of the liquid is higher than the surface tension (surface energy for solids) of the solid to be measured; otherwise, the liquid will spread on the solid surface. Since most organic and inorganic solids have a surface energy of around 40 mJ/m^2^, contact angle liquids must have a surface tension >40 mJ/m^2^ to be measurable.^28^ Therefore, the following liquids were chosen for our measurements: water, formamide, and diiodomethane; their corresponding surface energy components are summarized in Table S2. The droplet size was fixed at 2 μL, and an average of at least 10 values measured on different locations on the surface was recorded for each sample. Several methods exist for measuring the contact angle on powdered solid materials, such as biofoulants, including wicking techniques (Washburn method), the heat of immersion, inverse gas chromatography (IGC), captive bubble method, compressed pellets or discs, and the adhesive tape method.^29,30^ For BSA and HA, nonporous compacted discs cannot be obtained by pressing their powders even after grinding. Likewise, the adhesive tape method did not work either. Once placed on the porous BSA and HA powders, the liquid droplets instantly sank due to the capillarity caused by their high porosities. Alternatively, cleaned microscope slides (Corning, Inc.) were soaked in 2000 mg/L of BSA and HA solutions at 4 °C overnight. The next day, the slides were taken out of the solutions and their surfaces were wetted and covered with a reasonable amount of coating solutions and were then left to dry at ambient temperature. These coated slides were used for the contact angle measurements (Table 1).

**Table 1:** Summary of the Parameters Measured From Zeta Potential and Contact Angle Measurements^a^

	polymer film					model biofoulant	
parameter	CA	PVC	PVDF	PS		BSA	HA
ψ_*i*_ (mV)	–55.1	–32.9	–42.7	–52.9		–0.9	–47.2
θ_w_ (°)	55.7	61.4	78.9	68.3		41.4	19.3
θ_f_ (°)	76.6	75.6	72.1	75.6		44.6	36.2
θ_d_ (°)	43.1	31.6	58.8	31.9		39.7	39.8
γ_S_^LW^ (mJ/m^2^)	38.02	43.55	29.27	43.42		39.76	39.71
γ_S_^+^ (mJ/m^2^)	0.00	0.00	0.00	0.00		0.00	0.02
γ_S_^–^ (mJ/m^2^)	24.61	15.30	12.42	10.26		45.97	64.65
γ_S_^AB^ (mJ/m^2^)	0.00	0.00	0.00	0.00		0.00	2.33
γ_S_^TOT^(mJ/m^2^)	38.02	43.55	29.27	43.42		39.76	42.04
*A_ii_* (10^–20^) (J)	7.066	8.094	5.440	8.070		7.389	7.380

a The measurements were performed in DI water with an average pH value of 5.6.

### Zeta Potential Measurements of Model Biofoulants and Surface Zeta Potential Measurements of Polymer Films

7–8 mg of spheres/coated spheres was dispersed in 1.5 mL of DI water and sonicated before each measurement. A Zetasizer Nano ZS (Malvern) was used to measure the zeta potential of the spheres/coated spheres and the polymer films (Table 1). The electrophoretic mobilities of the spheres were obtained by performing electrophoresis experiments on the samples and measuring the velocity of the particles using a laser doppler velocimetry. The measured electrophoretic mobilities were then converted to zeta potentials using the Smoluchowski approximation. Similarly, zeta potential measurements of BSA (1.5 wt %) and HA (0.1 wt %, prepared in the same way as the probe coating solutions and then filtered) solutions were performed in DI water. In comparison, the surface zeta potentials of the polymer films were measured using the surface zeta potential cell (ZEN1020). Zeta potential measurements of the coated and uncoated spheres were carried out within the pH range of 3–11, while those for the polymer films were carried out across the pH range of 3–7.

### QCM-D Measurements

The dynamic adsorption behavior of BSA and HA onto the polymer films and the structure of the adsorbed fouling layers were investigated using QSense E4 QCM-D (Biolin Scientific, Sweden). The sensor surfaces were initially cleaned by rinsing the sensors with the following solvents in order: NMP, acetone, ethanol, and then DI water. The sensor surfaces were then dried with N_2_ gas. Following this step, the sensors were placed in a plasma cleaner (Diener electronic GmbH & Co. KG, Germany) for 20 min. The cleaned gold-coated sensor crystals (QSX 301 Au; Qsence) were spin-coated with the polymer solutions previously used to prepare the polymer films. Every gold-coated sensor was fixed on the center of the substrate holder of a spin coater (Laurell WS-650), and 10 μL (for CA and PVDF polymer solutions) or 20 μL (for PVC and PS polymer solutions) was dripped into the sensor surface. Low-/high-speed conditions were selected (a low speed of 1000 rpm for *t*_1_ = 15 s, followed by a high speed of 6000 rpm for CA and PVDF, 2000 rpm for PVC, or 4000 rpm for PS, for *t*_2_ = 40 s) to obtain a thin and homogeneous coating on the sensor surface.^31^ Due to the high viscosity of the PS solution used in PS film preparation, a diluted solution with a concentration of 100 g/L was used for QCM-D sensor coating. The coated sensors were then placed in an oven (Carbolite Gero Ltd.) and heated at 35 °C overnight. For the QCM-D measurements, the coated sensors were installed in the flow module and the system temperature was set at 21 °C. Initially, DI water was introduced as the background solution into the system until a stable baseline was obtained. Next, 50, 75, or 100 ppm BSA/HA solutions were injected into the system until equilibrium and the changes in frequencies and dissipation energies versus time were monitored. The BSA/HA solutions were prepared in the same way used to prepare the coating solutions for the colloidal AFM sphere probes. During the measurement, the flow rate was kept constant at 40 μL. Finally, desorption or rinsing was carried out by injecting DI water again into the system for 30 min.

## Modeling

### Analysis of the Adhesion Affinities between the Polymer Films and the Model Biofoulant-Coated AFM Probes

AFM retraction curves collected with the coated colloidal probes were analyzed individually. The adhesion was quantified from the retraction curves as the maximum rupture force in each curve (Figure S3). This represents the force that is needed to rupture the bond. The adhesion energy or the work of adhesion was computed as the area under the force–distance retraction curve with the baseline aligned at zero force (Figure S3). Generally, force and adhesion are related as^32^
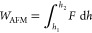
1where *W*_AFM_ is the adhesion energy obtained from the AFM retraction curves, *F* is the rupture force, and *h* is the separation distance. To quantify the adhesion energy, the integral in eq 1 was evaluated using the trapezoidal rule (eq 2):^32,33^
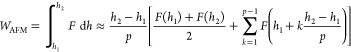
2where *h*_1_ and *h*_2_ are the first and last distance points at which the retraction curve crosses the zero–force axis (Figure S3) and *p* is the number of data points collected per retraction force curve in the integral interval, which varied from one curve to another. However, a uniform grid was always used in computing the integral in eq 1.

### Hansen Solubility Parameter Calculations

The Hansen solubility parameters for common organic solvents are already recorded in the Hansen database (HSPiP software).^24^ In our previous work,^23^ the Hansen sphere method was applied to determine the solubility parameters of the four polymer films (Table S3). The Hansen solubility parameters of BSA were taken from a previous work.^25^ The organic solvents were divided into good and poor solvents. The solvents marked as good solvents were given a score of 1, while the ones marked as bad solvents were assigned a score of 0 in the HSPiP software.

### Interfacial Surface Energy Calculations and Hamaker Constant Estimation

The surface energy components of the model biofoulants and the polymer films were determined using the contact angles measurements with three probe liquids, according to the extended Young’s equation (eq 5):^27,34^
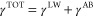
3
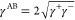
4
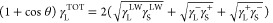
5where θ is the contact angle; γ_L_^TOT^, γ_L_^LW^, γ_L_^–^, and γ_L_^+^ are the total liquid surface tension, apolar Liftshitz–van der Waals component, and electron–acceptor and electron–donor parameters of the polar Lewis acid–base component (γ^AB^), respectively, and γ_S_^LW^, γ_S_^+^, and γ_S_^–^ are the surface energy components of the model biofoulants and the polymer films, which can be obtained from eq 5 (Table 1).

The combined Hamaker constant (*A*_132_) of the biofoulant (1), the polymer film (2), and the aqueous medium (3) can be approximated by^35^
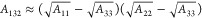
6where *A_ii_* is the individual Hamaker constant of materials 1, 2, and 3 and is given by^27^
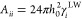
7where *h*_0_ is the minimum equilibrium distance between two parallel flat layers of material *i*, usually taken as 0.157 nm.^27^ γ^LW^ values of the model biofoulants and the polymer films in eq 7 can be determined from eq 5 using the OCG approach (Table 1). However, for porous solid powders, it is sometimes a complex procedure to make nonporous films to perform contact angle measurements. Since both surface energies and solubility parameters are decided by molecular interactions, there should be an intrinsic connection between the two. Several equations have been proposed to link the two quantities. However, these equations contain the molar volume as a parameter that is hard to obtain for solid materials.^36,37^ Thus, only molar volume-free empirical correlations in the literature were utilized to estimate γ^LW^ from Hansen solubility parameters obtained via dissolution tests. The first empirical equation, proposed by Jia and Shi, is^36^
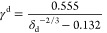
8where γ^d^ is the dispersive component of the surface energy and δ_d_ is dispersive solubility parameter. In this work, we treat γ^d^ in eq 8 as γ^LW^ in eqs 3, 5, and 7. The second empirical equation, proposed by Yu and Hou, is^37^
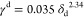
9

The obtained γ^d^ values were then used to calculate the individual Hamaker constants (*A_ii_*) according to eq 7. For the XDLVO analysis, γ^–^ and γ^+^ are required. According to van Oss, if the γ^–^ and γ^+^ of γ^AB^ cannot be determined separately, their product can be reported and the only way to obtain the interfacial surface energies is via the following extremely approximative equation:^38^
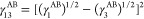
10

Using eq 10, one can derive the following equation for :
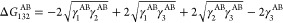
11

The AB component of the interfacial tension (γ^AB^) can be calculated as
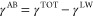
12where γ^TOT^ can be approximated by the empirical equation proposed by Yu and Hou, as^37^
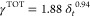
13

In addition, Δ*G*_132_^LW^ can be calculated using the following equation:
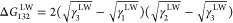
14

Finally, the total Δ*G*_132_^adh^ will be given by
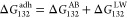
15

### DLVO and XDLVO Calculation of Interaction Energies and Forces

The DLVO and XDLVO analyses were applied to describe the interaction forces and energies between the model biofoulants and the polymer films. The DLVO theory describes the total interaction energy between a biofoulant and a polymer surface as the sum of attractive Lifshitz–van der Waals (LW) and attractive or repulsive electrostatic (EL) double-layer interaction energies:
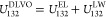
16where *U*_132_^DLVO^ is the total interaction energy between the polymer film (1) and the biofoulant (2) in water (3), *U*_132_^EL^ is the EL interaction term, and *U*_132_^LW^ is the nonpolar LW interaction term. The EL interaction energy between a flat surface and a sphere in an aqueous environment can be estimated using the linear superposition approximation (LSA) method, expressed as^39,40^
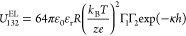
17
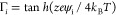
18
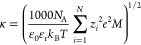
19where ε_0_ is the permittivity of vacuum (8.85 × 10^–12^ C^2^/(J·m)), ε_r_ is the relative permittivity of the solvent (78 for water), *R* is the radius of the sphere (2.5 × 10^–6^ m), *k*_B_ is the Boltzmann constant (1.380 × 10^–23^ J/K), *T* is the absolute temperature (298 K), *z* is the valence of bulk ions (+1 for Na^+^ and −1 for Cl^–^), *M* is the concentration of bulk ions (taken as 0.0027 M for DI water), *e* is the electron charge (1.602 × 10^–19^ C), Γ_1_ is the dimensionless surface potential of the polymer films/biofoulants, ψ_*i*_ is the surface potential of the polymer films/biofoulants (V), taken as the measured zeta potential for the surfaces involved,^34^ κ is the inverse Debye screening length (nm^–1^) calculated using eq 19, *h* is the separation distance between the biofoulant and the polymer film surface (nm), and *N*_A_ is Avogadro’s constant (6.022 × 10^23^ mol^–1^). The LW interaction energy between a flat surface and a sphere in an aqueous environment can be calculated using a retarded expression suggested by Gregory,^41^ given by
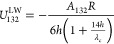
20where λ_c_ is the characteristic wavelength of the interactions, often assumed to be 100 nm.

The XDLVO theory introduces a third term to the total energy between the model biofoulants and the polymer films, i.e., the polar Lewis acid–base (AB) interaction energy, which accounts for hydrogen bonding interactions, hydrophobic interactions, steric interactions, and hydration interactions (hydrophilic repulsion).^26^ According to the XDLVO theory, the total interaction energy can be expressed as
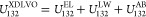
21where *U*_132_^AB^ is the polar AB interaction term. The AB interaction energy between a flat surface and a sphere in an aqueous environment can be attractive or repulsive and is expressed as follows:^42^
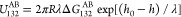
22where λ is the characteristic decay length of AB interactions in water (≈0.6 nm) and Δ*G*_132_^AB^ is the AB adhesion energy per unit area (J/m^2^) at the minimum equilibrium distance (*h*_0_). Thus, it can be calculated using the experimentally determined Lewis acid–base components of the surface energy (eq 5) for the biofoulants and polymer films, as follows:^34^
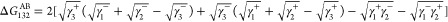
23

Since the interaction force (*F*) and energy (*U*) are related such as *F* = – dU/d*h*, the El, LW, and AB interaction forces between a flat surface and a sphere in an aqueous environment can be obtained by deriving the energy equations above, as follows:
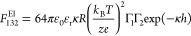
24
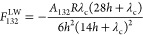
25
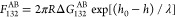
26

### Macroscopic Adhesion Free Energy Calculations between BSA/HA-Functionalized Colloidal Probes and Polymer Films in Water

The free energy of adhesion per unit area (Δ*G*_132_^adh^) between a flat surface and a sphere brought into contact in an aqueous environment can be approximated at the hypothetical minimum equilibrium cutoff distance (i.e., at *h*_0_) using a thermodynamic approach from the surface energy components, as follows:
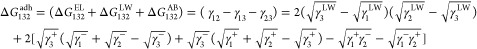
27
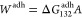
28where γ_*ij*_ is the interfacial surface energy between materials *i* and *j*, *W*^adh^ is the macroscopic adhesion energy estimated using the macroscopic contact angle measurements (mJ), and *A* is the contact area in m^2^ between the AFM colloidal probe and the polymer film surface. The term Δ*G*_132_^EL^ usually is negligible at contact distances (*h*_0_) and thus is neglected.^43^ If Δ*G*_132_^adh^ < 0, the interactions are attractive and surface biofouling is spontaneous, while if Δ*G*_132_^adh^ > 0, the interactions are repulsive and additional energy will be required for surface biofouling to occur. The contact area in eq 28 was estimated as , where *a*_0_ is the contact radius calculated based on the Johnson–Kendall–Roberts (JKR) contact model of mechanics, as follows:^44^
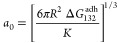
29
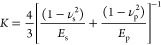
30where *a*_0_ is the contact radius at zero applied load and which was chosen because the corresponding *W*_AFM_ was calculated starting from *h*_1_ at which the retraction curve crosses the zero–force axis (Figure S3), *R* is the AFM spherical probe radius (2.5 × 10^–6^ m), Δ*G*_132_^adh^ is the free energy of adhesion per unit area calculated using eq 27, *K* is the reduced Young’s modulus of the probe–polymer film system, and ν_s_ and ν_p_ are the Poisson’s ratios of silica (0.17)^45^ and the polymer films (CA: 0.43,^46^ PVC: 0.375,^47^ PVDF: 0.34,^47^ PS: 0.37^47^), respectively. *E*_s_ and *E*_p_ are the Young’s moduli of silica (70 GPa)^45^ and the polymer films (CA: 2 GPa,^48^ PVC: 2.8G Pa,^48^ PVDF: 2.85 GPa,^49,50^ PS: 2.6 GPa^48^), respectively. Furthermore, the maximum number of the interacting biofoulant molecules was predicted by dividing the contact area (*A*) for each polymer film estimated using the JKR theory over the area occupied by one biofoulant molecule (π*r*^2^), where *r* is the radius of a biofoulant molecule.^51^ The hydrodynamic diameter of the BSA molecule is around 7 nm (*r*_BSA_ ≈ 3.5 nm),^52^ and that of HA is in the range of 2.3 to 9.0 nm (*r*_HA_ ≈ 5.65 nm).^53^

### Adsorbed Mass and Standard Gibbs Free Energy Calculations from QCM-D Measurements

The model biofoulant deposition or adsorption experiments were performed on clean QCM-D sensors each spin-coated with one of the four polymer films. Both the frequency (Δ*f*) and the dissipation (Δ*D*) of the coated crystal sensor at the third overtone were collected in real time. The dissipation of the sensor is the energy loss per oscillation cycle when the driving power of the oscillation is switched off, divided by the total energy stored in the system, and it is directly related to the viscoelasticity of the deposited film. The deposition or adsorption of BSA/HA on the coated sensor surface resulted in an increase in mass, which was recorded as a decrease in the crystal sensor frequency (Δ*f*). The relationship between Δ*f* and Δ*m* for rigid films for which Δ*D* is close to zero can be described by the Sauerbrey equation:^54^
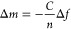
31where Δ*m* is the change in mass, *C* is the mass sensitivity constant of the sensor (17.7 ng Hz^–1^ cm^–2^ for a 4.95 MHz crystal), *n* is the overtone number, and Δ*f* is the shift in frequency. Reviakine et al. suggested that if Δ*D_n_*/( – Δ*f_n_*/*n*) ≪ 4 × 10^–7^ Hz^–1^ for a 5 MHz crystal, then the film is rigid and the Sauerbrey equation can be used.^55^ The rate of adsorption was determined from the initial slope of the frequency shift at a given time, according to^56^
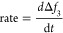
32

In addition, the ratio of |Δ*D*/Δ*f*| can provide information on the structural properties of the adsorbed layer. A lower value of |Δ*D*/Δ*f*| suggests the formation of a dense and compact structure, while a higher value indicates the formation of a more viscoelastic layer with a soft and open structure.^16,57,58^ A plot of Δ*D* versus Δ*f* during the adsorption process shows the dynamic change of the adsorbed layer structure, and multiple phases can suggest changes in molecular conformation or orientation.^16^ In contrast, Δ*D* versus Δ*f* at adsorption equilibrium illustrates the structure of the formed layer at equilibrium. For more information on QCM-D, the reader is referred to a review by Easley et al.^59^

The Langmuir isotherm is one of the most widely used models in the literature to describe the adsorption equilibrium process. The Langmuir isotherm assumes that monolayer adsorption takes place on a homogeneous surface with negligible interaction forces between the adsorbed molecules. The Langmuir isotherm model can be expressed as^60^
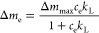
33where Δ*m*_e_ is the change in mass (ng/cm^2^) at equilibrium obtained from QCM-D measurements, Δ*m*_max_ is a constant that stands for the maximum change in mass (ng/cm^2^) or the maximum adsorption capacity, *c*_e_ is the equilibrium concentration of BSA in the solution (ppm or mg/L), and *k*_L_ is the Langmuir isotherm constant (L/mg), which represents the ratio of the adsorption rate to the desorption rate and which is related to the affinity of BSA with the polymer films. A plot of *c*_e_*/*Δ*m*_e_ versus Δ*m*_e_ should yield a straight line and the values of *k*_L_ and Δ*m*_max_ can be obtained from the slope (1/Δ*m*_max_) and intercept (1/(*k*_L_Δ*m*_max_)).

In addition, the standard Gibbs free energy change of adsorption (Δ*G*_ads_^°^) is a measure of the thermodynamic driving force that describes the adsorption potential of a protein toward a surface. Δ*G*_ads_^°^ can be estimated from^61^
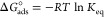
34where *R* is the universal gas constant (8.314 J/mol·K), *T* is the temperature (K), and *K*_eq_ is the dimensionless equilibrium constant, which can be approximated by multiplying the Langmuir constant (*k*_L_) by the molecular weight of BSA (66,430.0 g/mol), 1000, and then 55.5.^62^ The sign of Δ*G*_ads_^°^ gives an indication of the direction of the reaction or process. If Δ*G*_ads_^°^ < 0, adsorption is favored, whereas if Δ*G*_ads_^°^ > 0, desorption is favored. Furthermore, to compare Δ*G*_ads_^°^ (in J/mol) with the AFM adhesion energies (in aJ), Δ*G*_ads_^°^ can be multiplied by the number of BSA moles (no. of BSA molecules divided by Avogadro’s number) predicted in the contact area and which was determined by the JKR model in the AFM studies.

### Statistical Description of Data

Statistical tests were used to determine whether the AFM adhesion forces and energies measured between model biofoulants and polymer films in DI water had statistically significant differences between the four polymer films and between the biofoulants BSA and HA. The nonparametric Kruskal–Wallis ANOVA and the nonparametric Mann–Whitney tests available in OriginPro 2020 SR1 were applied to the multiple-group data and the two-group data, respectively. A standard sign convention for the interaction forces and energies was used in this context, whereby a negative sign indicated attraction and a positive sign meant repulsion.

## Results and Discussion

### Coating Amine-Functionalized Silica Spheres with Model Biofoulants

**Figure 1:**
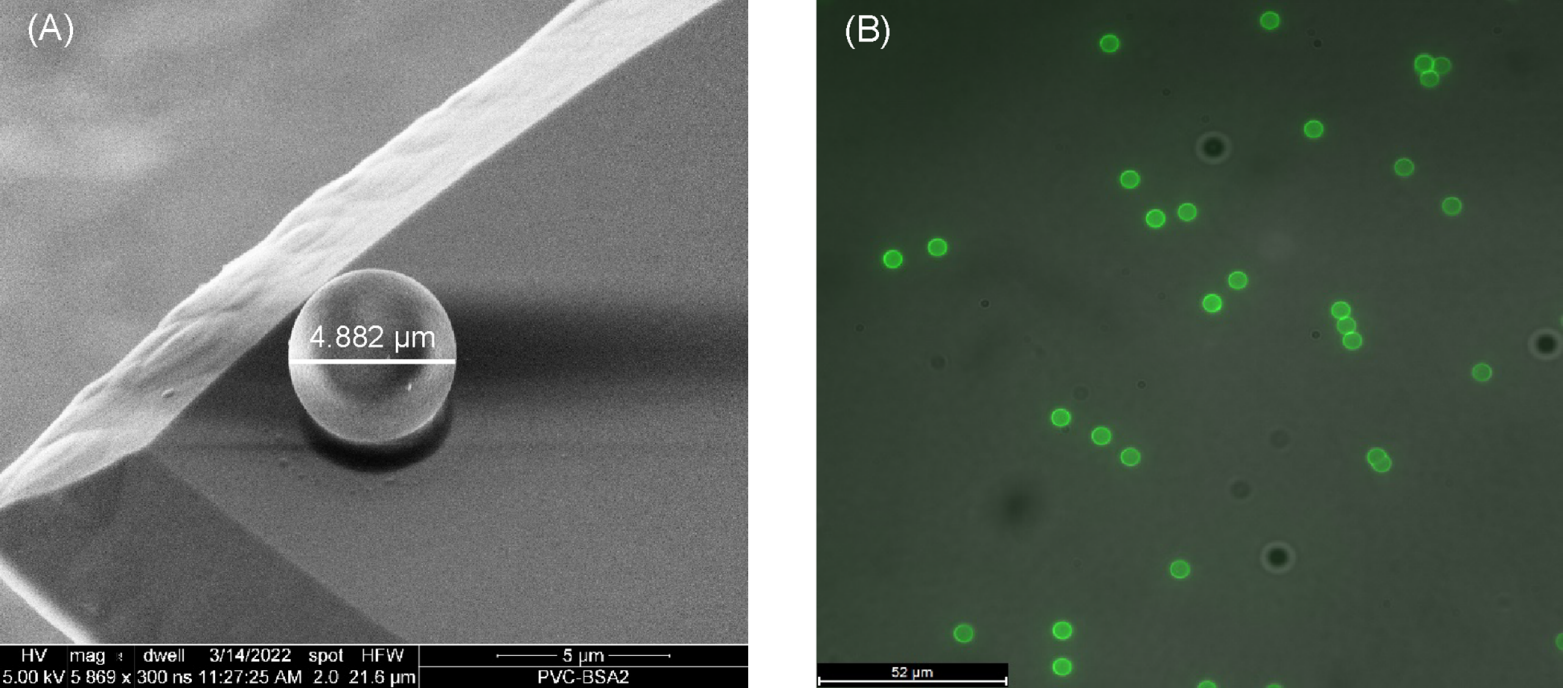
(A) SEM image of a BSA-coated colloidal AFM probe. (B) Fluorescence microscopy image of a FITC-labeled BSA-coated amine-functionalized silica spheres that were used in preparing colloidal probes.

Figure 1A shows a BSA-coated sphere with a diameter of 4.882 μm glued to the apex of a tipless cantilever. The fluorescent layer on the surface of the spheres, displayed in Figure 1B, ensured that a successful BSA coating was assembled on the surface of the spheres and can be later used to probe the interactions between BSA molecules and the polymer films. Although the fluorescence image cannot ensure the full coverage of biofoulants on the silica sphere surface due to the limited resolution of the microscope, it can show that a successful and uniform coating was attained. A control sample of uncoated amine-functionalized silica spheres was also imaged, where no fluorescence signal was shown (Figure S1A). The fluorescent layer on the probe appeared even after its use in AFM force measurements, confirming the stability of the coating during the course of the measurement (S1B). In addition, a change was noticed in the zeta potentials as a function of pH after coating, demonstrating the accomplishment of the coating process. Initially, BSA and HA molecules in DI water (pH ≈ 5.6) had zeta potentials of −18.3 and – 47.6, respectively. However, due to coating with the negatively charged BSA or HA, the negative charges on the positively charged amine-functionalized spheres (+46.7 mV) increased and their zeta potentials were thus reduced (Figure S4).

### Adhesion Forces and Energies Measured between BSA/HA-Functionalized Colloidal Probes and Polymer Films in Water by AFM

**Figure 2:**
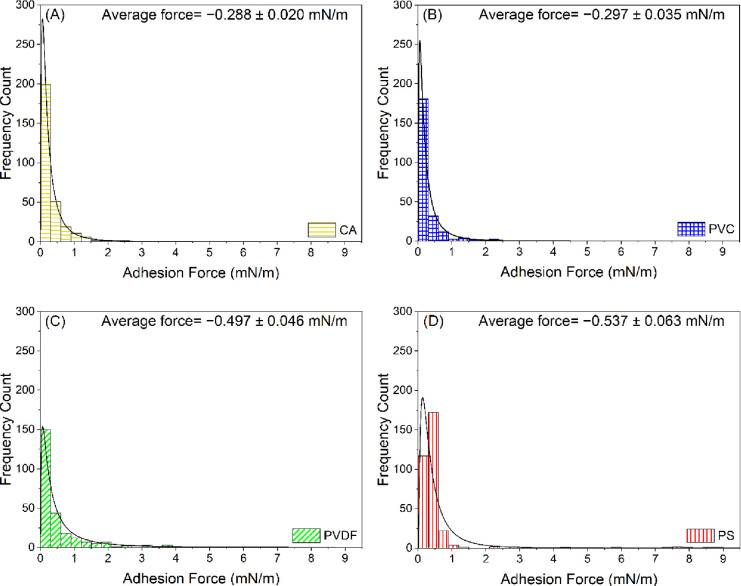
Histograms showing the distribution of normalized adhesion forces (mN/m) quantified between BSA-coated colloidal probes and (A) CA, (B) PVC, (C) PVDF, and (D) PS in DI water. Solid lines in the histograms represent the log-normal distribution function fits to the adhesion force data. Errors reported in the figures are the standard error of the mean. The adhesion forces measured by AFM are negative in sign (attractive). However, absolute values of the adhesion forces are plotted here for clarity. CA: cellulose acetate, PVC: polyvinyl chloride, PVDF: polyvinylidene fluoride, and PS: polysulfone.

**Figure 3:**
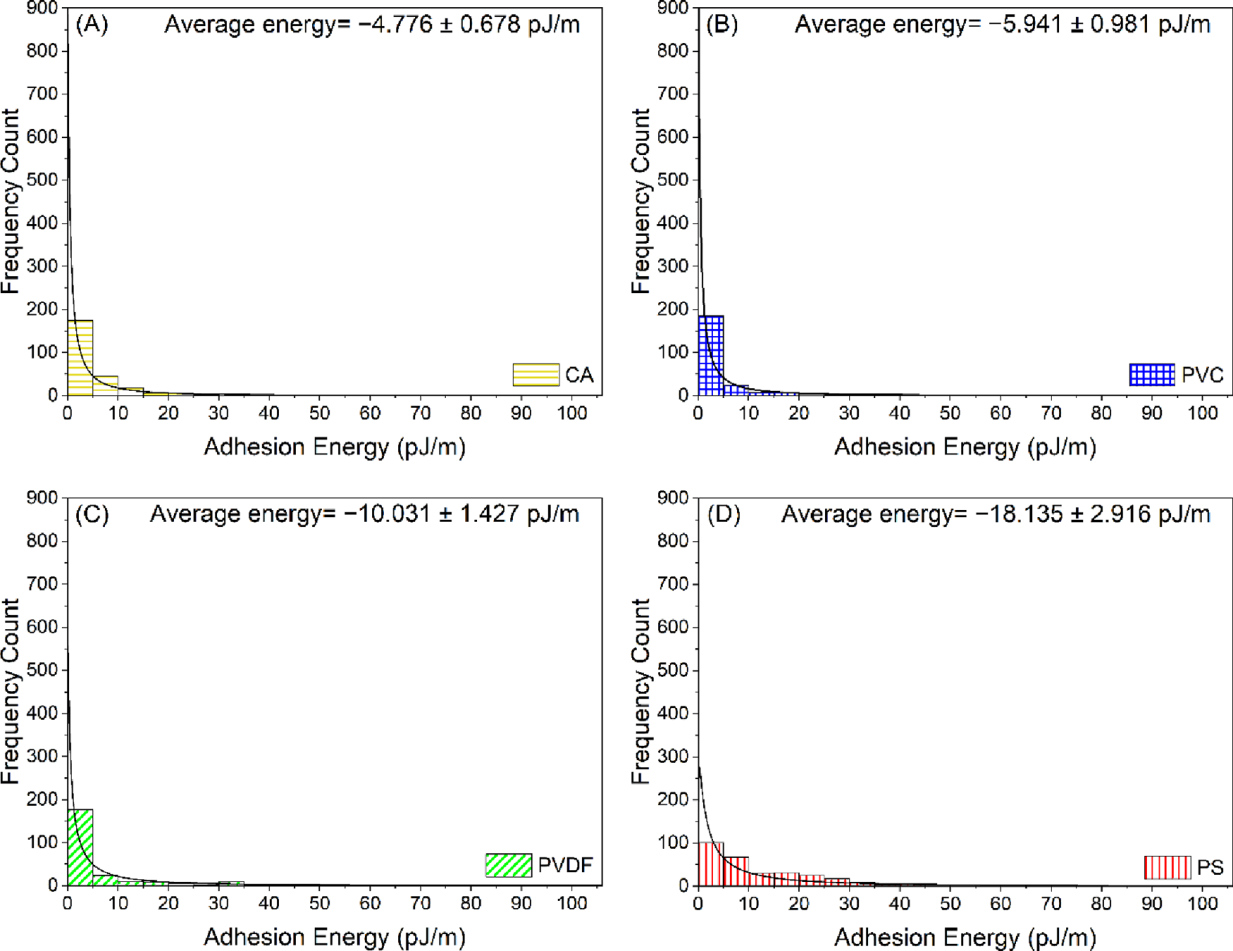
Histograms showing the distribution of normalized adhesion energies (pJ/m) quantified between BSA-coated colloidal probes and (A) CA, (B) PVC, (C) PVDF, and (D) PS in DI water. Solid lines in the histograms represent the log-normal distribution function fits to the adhesion energy data. Errors reported in the figures are the standard error of the mean. The adhesion energies measured by AFM are negative in sign (attractive). However, absolute values of the adhesion energies are plotted here for clarity. CA: cellulose acetate, PVC: polyvinyl chloride, PVDF: polyvinylidene fluoride, and PS: polysulfone.

**Figure 4:**
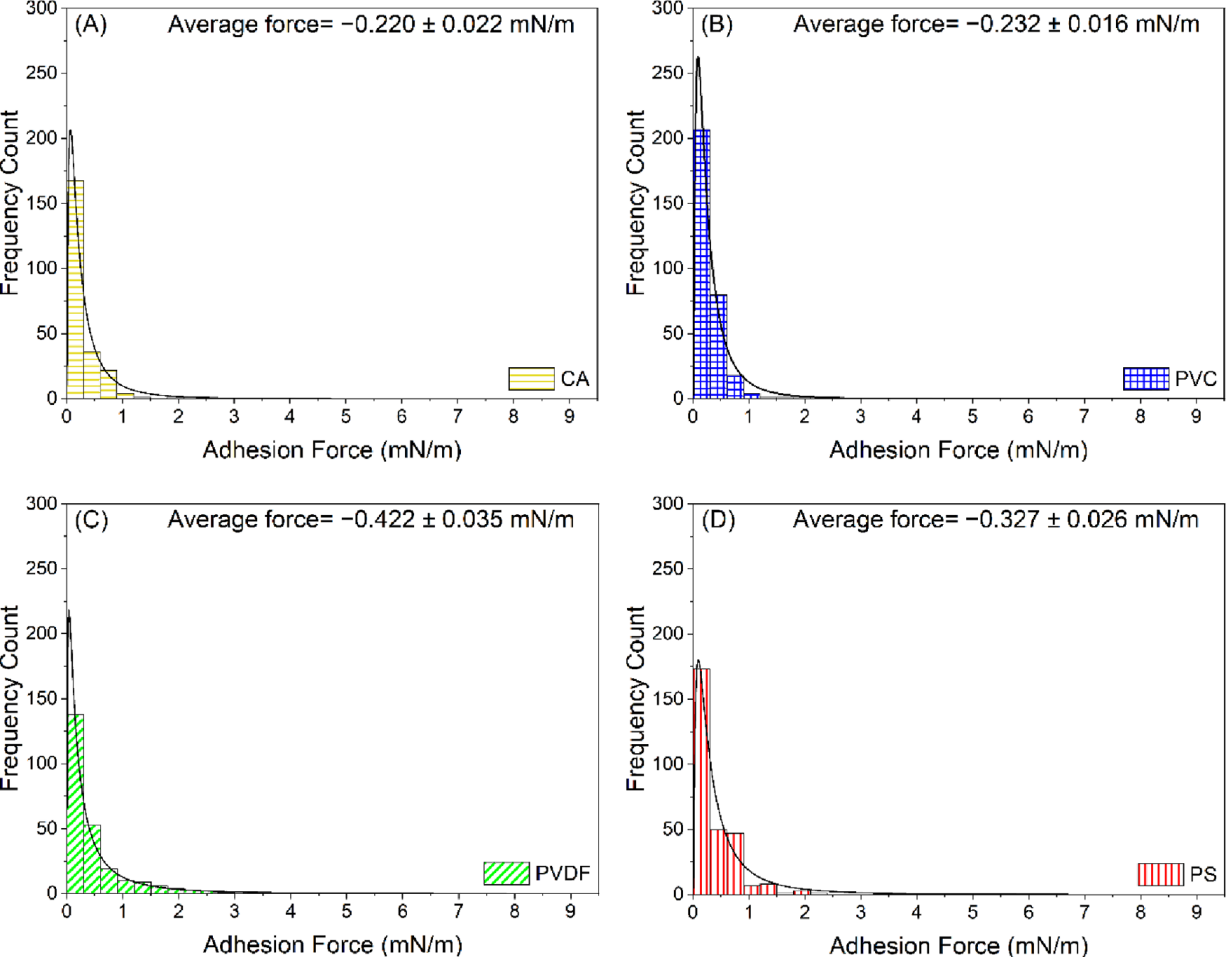
Histograms showing the distribution of normalized adhesion forces (mN/m) quantified between HA-coated colloidal probes and (A) CA, (B) PVC, (C) PVDF, and (D) PS in DI water. Solid lines in the histograms represent the log-normal distribution function fits to the adhesion force data. Errors reported in the figures are the standard error of the mean. The adhesion forces measured by AFM are negative in sign (attractive). However, absolute values of the adhesion forces are plotted here for clarity. CA: cellulose acetate, PVC: polyvinyl chloride, PVDF: polyvinylidene fluoride, and PS: polysulfone.

The distributions of the adhesion forces and the adhesion energies quantified between the BSA-coated AFM colloidal probes and the four polymer films are represented in Figures 2 and 3, respectively. The distributions of the adhesion forces quantified between the HA-coated AFM colloidal probes and the four polymer films are represented in Figure 4. To illustrate the distribution of the data shown in Figures 2–4, the mean, median, range, and standard deviation values were computed (Table 2). The highest mean values of the normalized adhesion forces (−0.537 mN/m) and adhesion energies (−18.135 pJ/m) were observed for PS in comparison to the other polymer films. The mean value of the normalized adhesion forces quantified for PS was 46, 45, and 8% higher than the mean values obtained for CA, PVC, and PVDF, respectively (Table 2). Similarly, the mean value of the normalized adhesion energies quantified for PS was 74, 67, and 45% higher than the mean values obtained for CA, PVC, and PVDF, respectively.

**Table 2:** Summary of the Normalized Adhesion Forces and Adhesion Energies Quantified between BSA-Coated AFM Colloidal Probes and the Four Polymer Films in Water.^a^

		polymer film			
model biofoulant	measured parameter	CA	PVC	PVDF	PS
BSA		Normalized adhesion forces (*F*_AFM_/*R*)			
	mean (mN/m)	–0.288	–0.297	–0.497	–0.537
	standard dev. (mN/m)	0.329	0.538	0.725	1.136
	median (mN/m)	–0.190	–0.142	–0.195	–0.374
	range (mN/m)	1.921	4.235	3.756	8.911
	standard error of the mean (mN/m)	0.020	0.035	0.046	0.063
	no. of curves	276	241	253	328
		Normalized adhesion energies (*E*_AFM_/*R*)			
	mean (pJ/m)	–4.776	–5.941	–10.031	–18.135
	standard dev. (pJ/m)	10.762	15.228	22.740	50.342
	median (pJ/m)	–1.797	–1.295	–1.195	–8.438
	range (pJ/m)	148.095	108.765	142.006	663.887
	standard error of the mean (pJ/m)	0.678	0.981	1.427	2.916
	no. of curves	252	241	254	298
HA		Normalized adhesion forces (*F*_AFM_/*R*)			
	mean (mN/m)	–0.220	–0.232	–0.422	–0.327
	standard dev. (mN/m)	0.332	0.281	0.553	0.435
	median (mN/m)	–0.0767	–0.116	–0.205	–0.128
	range (mN/m)	2.792	2.23	2.874	3.402
	standard error of the mean (mN/m)	0.022	0.016	0.035	0.026
	no. of curves	233	313	245	291

a The measurements were performed in DI water with an average pH value of 5.6. In addition, the normalized adhesion forces quantified between HA-coated AFM colloidal probes and the four polymer films in water are also listed.

The nonparametric Kruskal–Wallis ANOVA test indicated that the variations in the adhesion force and the adhesion energy values acquired for BSA were statistically significant among the polymer films (*P* < 0.05). For BSA, the trends of the mean values of the adhesion forces and the adhesion energies observed for the four polymer films were similar to each other. However, for HA, the highest mean value of the normalized adhesion forces (−0.422 mN/m) was observed for PVDF in comparison to the other polymer films. The mean value of the normalized adhesion force quantified for PVDF was 48, 45, and 22% higher than the mean values obtained for CA, PVC, and PS, respectively. The nonparametric Kruskal–Wallis ANOVA test indicated that the variations in the adhesion force values acquired for HA were statistically significant among the polymer films (*P* < 0.05). In comparison, the nonparametric Mann–Whitney test indicated that the variations in the adhesion force values were statistically significant between BSA and HA in pairs for each polymer film (*P* < 0.05), except for PVC and PVDF. The mean values of the normalized adhesion forces quantified between BSA and CA, PVC, PVDF, or PS were 24, 22, 15, and 39, respectively, higher than the corresponding mean values obtained for HA. This can be ascribed to the higher negative charge of HA molecules compared to BSA molecules and to the higher hydrophilic repulsion between HA and the polymer films (higher γ_S_^–^) compared to BSA (Table 1). These factors altogether are anticipated to produce a higher repulsive barrier and reduce the contact area resulting in lower adhesion strengths. Another possible reason can be the ability of the BSA molecules to form hydrophobic interactions with the polymer films, which is relatively higher than that of HA molecules due to the more hydrophobic nature of BSA molecules as deduced from their higher water contact angles (Table 1).

Our findings agree with previous results reported in the literature. For instance, a recent study by Wang et al. has reported that BSA caused more severe fouling to a PVDF membrane, faster adsorption rate, and higher irreversibility than alginate and HA in the presence of both Ca^2+^ and Na^+^ ions. This was attributed to the BSA’s stronger interaction forces with the membrane and the denser-formed fouling layer.^12^ Another study by Sun and Chen has shown that blending polyethersulfone with CA improved the hydrophilicity and the BSA antifouling properties of the membrane compared to the pure polyethersulfone membrane.^63^

### DLVO and XDLVO Model Predictions of the Adhesion Forces and Interaction Energies Interplaying between BSA/HA-Functionalized Colloidal Probes and Polymer Films

The surface charges of all the investigated polymer films were found to be negative and in the order of CA > PS > PVDF > PVC (Table 1). In addition, the individual Hamaker constant (*A_ii_*), the combined Hamaker constant (*A*_132_), and consequently, the Lifshitz–van der Waals interactions were found to be in the order of PVC > PS > CA > PVDF (Tables 1 and 3). The polymer films and the model biofoulants were found to be monopolar, except HA, which had a small value of γ_S_^+^. Comparable values of γ_S_^+^ and γ_S_^–^ have been reported in the literature for polymeric membranes,^34^ BSA,^64^ and HA.^65^ The γ_S_^–^ of the polymer films were found to be in the order of CA > PVC > PVDF > PS (Table 1).

**Table 3:** Summary of the Estimated Parameters between the Polymer Films and Both Model Biofoulants: BSA and HA in Water^a^

		polymer film			
parameter	model biofoulant	CA	PVC	PVDF	PS
*A*_132_ (10^–21^) (J)	BSA	4.553	5.871	2.254	5.841
	HA	4.542	5.856	2.249	5.826
Δ*G*_132_^LW^ (mJ/m^2^)	BSA	–4.90	–6.32	–2.43	–6.29
	HA	–4.89	–6.30	–2.42	–6.27
Δ*G*_132_^AB^ (mJ/m^2^)	BSA	16.58	5.98	2.07	–1.17
	HA	29.33	19.04	15.24	12.09
Δ*G*_132_^adh^ (mJ/m^2^)	BSA	11.68	–0.34	–0.36	–7.46
	HA	24.45	12.74	12.82	5.82
*a*_0_(nm)^b^	BSA	–75.76	21.10	21.71	61.09
	HA	–96.91	–70.81	–71.61	–56.24
no. of biofoulant molecules^a^	BSA	–468.6	36.4	38.5	304.6
	HA	–294.2	–157.0	–160.6	–99.1

a The measurements were performed in DI water with an average pH value of 5.6.

b The negative signs of the contact radius (a_0_) and the no. of biofoulant molecules resulted from repulsive  values.

At the closest distance of contact (≈ 0.157 nm), the electrostatic forces (*F*^El^) were found to be repulsive and relatively negligible (Table 4). The electrostatic interactions between the polymer films and HA molecules were higher than those with BSA molecules, which is to be expected due to the higher negative charge of the HA molecules. It should be noted that the zeta potential of BSA molecules was characterized as −18.3 mV, whereas BSA-coated spheres had a zeta potential of −0.9 mV. The initial zeta potential of the uncoated amine-functionalized silica spheres was 46.7 mV. Similarly, HA molecules were characterized by a zeta potential of −47.6 mV, while HA-coated spheres had a zeta potential of −47.2 mV. The electrostatic repulsion was also seen in the approach curves collected during the AFM force measurements, which were performed in DI water. A control measurement was performed in 0.5 M NaCl, and the repulsion disappeared due to the screening effect and subsequent shrinkage (in 0.5 M NaCl, 1/κ ≈ 0.43 nm, and in DI water (0.0027 M), 1/κ ≈ 5.8 nm) of the electrostatic double layer (Figure S5). In comparison, the AFM approach curve obtained in DI water had a Debye length of around 70 nm, suggesting that the exact ionic strength of DI water is of the order of 10^–5^ M. Compared to the electrostatic forces, Lifshitz–van der Waals forces (*F*^LW^) were attractive between both model biofoulants: BSA and HA, and all the investigated films (Table 4). On the other hand, the Lewis acid–base interfacial interactions (Δ*G*_132_^AB^) and/or forces (*F*^AB^) were found to be repulsive for all of the polymer–biofoulant pairs, except for the BSA–PS pair (Tables 3 and 4).

**Table 4:** Theoretically Predicted DLVO and XDLVO Forces between the Polymer Films and the Model Biofoulants: BSA and HA in Water^a^^*,*^^b^

		polymer film			
parameter	model biofoulant	CA	PVC	PVDF	PS
*F*_132_^El^ (nN)	BSA	0.164	0.104	0.132	0.159
	HA	8.04	5.08	6.45	7.77
*F*_132_^LW^ (nN)	BSA	–76.93	–99.19	–38.03	–98.68
	HA	–76.74	–98.95	–37.99	–98.44
*F*_132_^DLVO^ (nN)	BSA	–76.76	–99.09	–37.95	–98.52
	HA	–68.70	–93.87	–31.54	–90.67
*F*_132_^AB^ (nN)	BSA	260.41	93.94	32.49	–18.44
	HA	460.76	299.07	239.39	189.92
*F*_132_^XDLVO^ (nN)	BSA	183.64	–5.15	–5.46	–116.97
	HA	392.07	205.21	207.85	99.25

a DLVO*/*XDLVO forces were calculated at the theoretically closest separation distance of 0.157 nm.

b The measurements were performed in DI water with an average pH value of 5.6.

The net interfacial interactions (Δ*G*_132_^adh^), assuming negligible electrostatic interactions, were found to be attractive between BSA and all of the polymer films except for CA. However, it was repulsive between HA and all of the polymer films. As mentioned earlier, a negative Δ*G*_132_^adh^ means that the adhesion is spontaneous, while a positive Δ*G*_132_^adh^ indicates repulsive interactions and that additional energy is required for the adhesion to occur. Since we have noticed adhesion events in the AFM force measurements between all of the polymer films and the model biofoulants, as well as detectable shifts in the frequency in the QCM-D adsorption measurements (as will be discussed later), we believe that this could have happened due to an overestimation of the γ_S_^–^ parameter determined from contact angles measured on biofoulant-coated glass slides, especially since the glass slides were not treated before the deposition of the biofoulant. In comparison, when the AFM probes were prepared, the biofoulants were coated on positively charged amine-functionalized silica spheres, thus resulting in a more uniform coating. The γ_S_^–^ of glass (SiO_2_) is approximately (52–54 mJ/m^2^),^56,66^ which is very close to the values we obtained for BSA and HA (Table 1). Furthermore, a critical γ_S_^–^ value was estimated at which the total energy (Δ*G*_132_^adh^) becomes repulsive, i.e., when γ_S_^–^ of the surface exceeds that critical certain value.^67^ For BSA, assuming γ_BSA_^LW^ is between 30 and 40 mJ/m^2^, the critical γ_S_^–^ was found to be between 12.825 and 14.868 mJ/m^2^. For HA, assuming γ_HA_^LW^ is between 30 and 40 mJ/m^2^, the critical γ_S_^–^ was found to be between 5.016 and 6.359 mJ/m^2^. Clearly, BSA has a lower γ^–^, so the polymer surface should have a higher γ^–^ than the values obtained for HA in order to repel the biofoulants. Therefore, the system should be analyzed to find the biofoulant with the lowest γ^–^ and then analyzed for the critical γ^–^.

**Figure 5:**
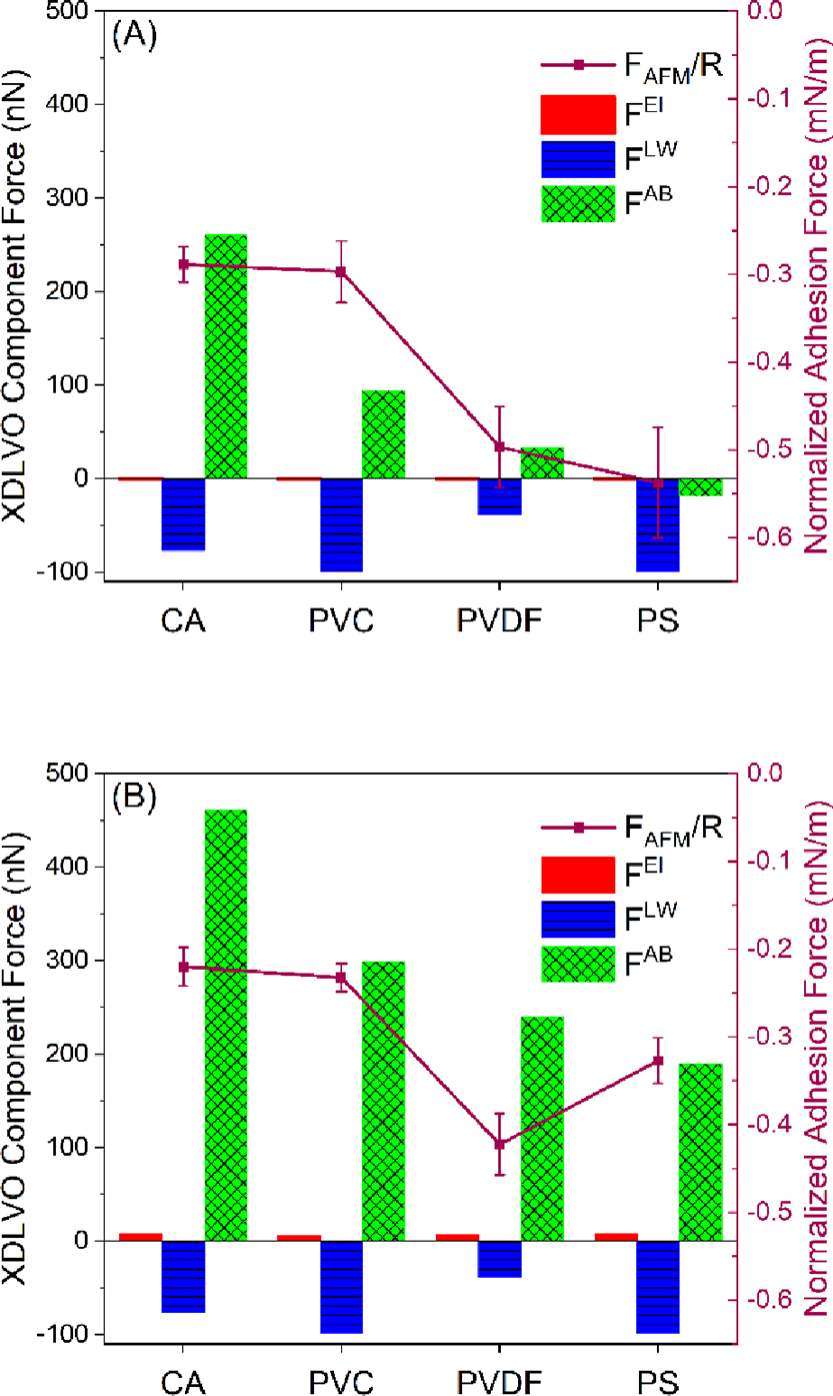
The contribution of XDLVO force components to the AFM adhesion force quantified between (A) BSA-coated colloidal probes or (B) HA-coated colloidal probes and the four polymer films in water. Errors reported in the figures are the standard error of the mean. CA: cellulose acetate, PVC: polyvinyl chloride, PVDF: polyvinylidene fluoride, and PS: polysulfone.

The adhesion force measured by AFM represents the net of three primary forces: the electrostatic forces, the Lifshitz–van der Waals forces, and the Lewis acid–base forces. The contribution of each XDLVO component force (*F*^El^, *F*^LW^, and *F*^AB^) to the normalized overall adhesion force measured by AFM is plotted in Figure 5. It is clear that AB interactions governed the adhesion of BSA to the CA film, while it was dominated by the attractive LW interactions for PVC, PVDF, and PS. In contrast, HA adhesion to all the polymer films was governed by the AB interactions. A similar trend was observed between the BSA-normalized adhesion forces (*F*_AFM_/*R*) and the sum of the XDLVO component forces (*F*^XDLVO^), calculated at the minimum equilibrium distance of 0.157 nm (Table 4, Figure 5). However, no trend was observed between the BSA-normalized adhesion forces (*F*_AFM_/*R*) and the sum of the DLVO component forces (*F*^DLVO^). Similarly, a trend was observed between the BSA-normalized adhesion energies (*W*_AFM_/*R*) and the sum of the XDLVO component energies (*U*^XDLVO^), calculated at the minimum equilibrium distance of 0.157 nm (data and plots not shown). However, no trend was observed between the BSA-normalized adhesion energies (*W*_AFM_/*R*) and the sum of the DLVO component energies (*U*^DLVO^). This suggests that XDLVO theory is more predictive of our system. When the normalized adhesion forces of BSA were plotted versus each XDLVO component force (*F*^El^, *F*^LW^, and *F*^AB^), *R*^2^ values were found to be 0.07, 0.08, and 0.71, respectively (Figure S6). This can demonstrate the controlling role of the AB interactions in the adhesion of BSA to the polymer films. This can be reflected also by observing that the highest adhesion was found for the polymer with the lowest γ^–^ and vice versa (Table 1). A higher γ^–^ will lead to a higher binding energy with water and increase the hydrophilic repulsion between the polymer surfaces and the biofoulants.^27^

In the case of HA, when the PVDF film was excluded, a similar trend was observed between the normalized adhesion forces (*F*_AFM_/R) and the sum of the XDLVO component forces (*F*^XDLVO^) for the three remaining films calculated at the minimum equilibrium distance of 0.157 nm (Table 4). However, even by excluding PVDF, no trend was observed between the HA normalized adhesion forces (*F*_AFM_/*R*) and the sum of the DLVO component forces (*F*^DLVO^). When the normalized adhesion forces of HA were plotted against each of the XDLVO component forces (*F*^El^, *F*^LW^, and *F*^AB^) excluding PVDF, *R*^2^ values were found to be 0.10, 0.33, and 0.74, respectively (Figure S7). Again, this can demonstrate the dominant role of the AB interactions in the adhesion of HA to the polymer films. The highest roughness observed for the PVDF film compared to other films can be one reason why it was an outlier (Table S1).

**Figure 6:**
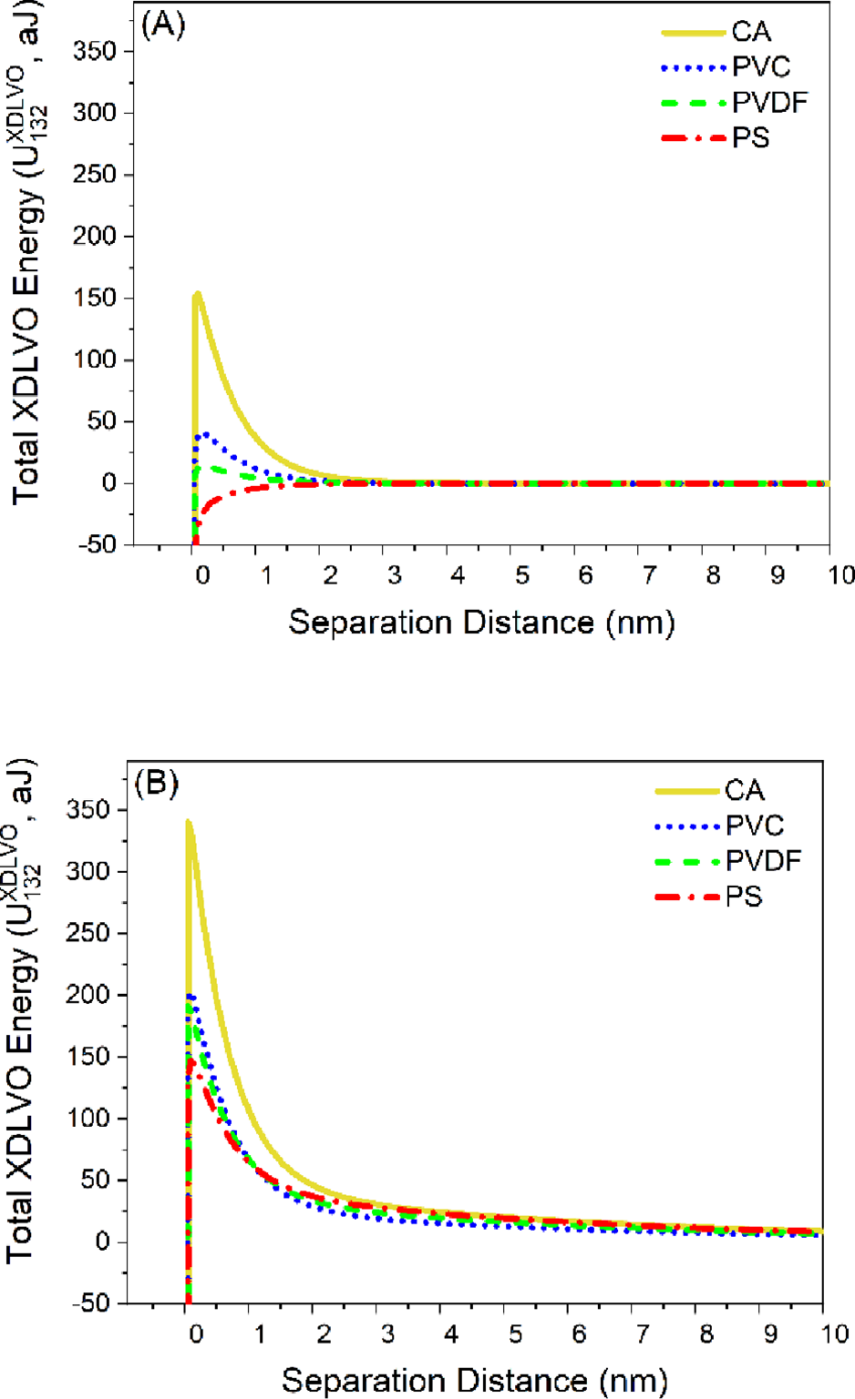
Total XDLVO energy profiles calculated between (A) BSA-coated colloidal probes or (B) HA-coated colloidal probes and the four polymer films in water. CA: cellulose acetate, PVC: polyvinyl chloride, PVDF: polyvinylidene fluoride, and PS: polysulfone.

Minimal energy barriers are generally believed to favor the adhesion to surfaces. Therefore, we analyzed the DLVO and XDLVO energy profiles between BSA/HA-coated colloidal probes and the four polymer films in water (Figure 6). A qualitative agreement was observed in the trends of the AFM adhesion energies and the XDLVO energy barriers or the AFM adhesion forces and the XDLVO energy barriers. In contrast, no trend was observed with DLVO energy barriers (plots not shown). In the case of HA, when the PVDF film was excluded, a qualitative agreement was also observed in the trends of the AFM adhesion forces (energies were not calculated) and the XDLVO energy barriers and no trend was observed with DLVO energy barriers. For BSA, when the total energy barriers were decoupled into their component energies, the highest share was found to be for AB interactions among the polymer films (95.5%, on average), followed by El (1.9%, on average) and then by LW (22.3%, on average) energies (Table 5). For HA, the highest share was noticed for AB interactions (92.2%, on average), followed by El (20%, on average) and then by LW (12.2%, on average) energies (Table 5).

**Table 5:** Theoretically Predicted XDLVO Energy Barriers between the Polymer Films and Both Model Biofoulants: BSA and HA in Water^a^

		polymer film			
parameter	model biofoulant	CA	PVC	PVDF	PS
*U*_132_^max^ (aJ)	BSA	154.07	41.72	14.41	no barrier
	HA	340.64	203.20	191.41	147.16
*U*_132_^El^ (aJ)	BSA	0.97	0.60	0.77	no barrier
	HA	47.79	29.94	38.34	45.80
*U*_132_^LW^ (aJ)	BSA	–18.71	–15.25	–5.85	no barrier
	HA	–37.59	–24.06	–18.61	–23.94
*U*_132_^AB^ (aJ)	BSA	171.81	56.36	19.50	no barrier
	HA	330.43	197.33	171.68	125.31

a The measurements were performed in DI water with an average pH value of 5.6.

### Dynamic Adsorption and Desorption Behavior of BSA and HA onto Polymer Films and Structure of Adsorbed Layers

**Figure 7:**
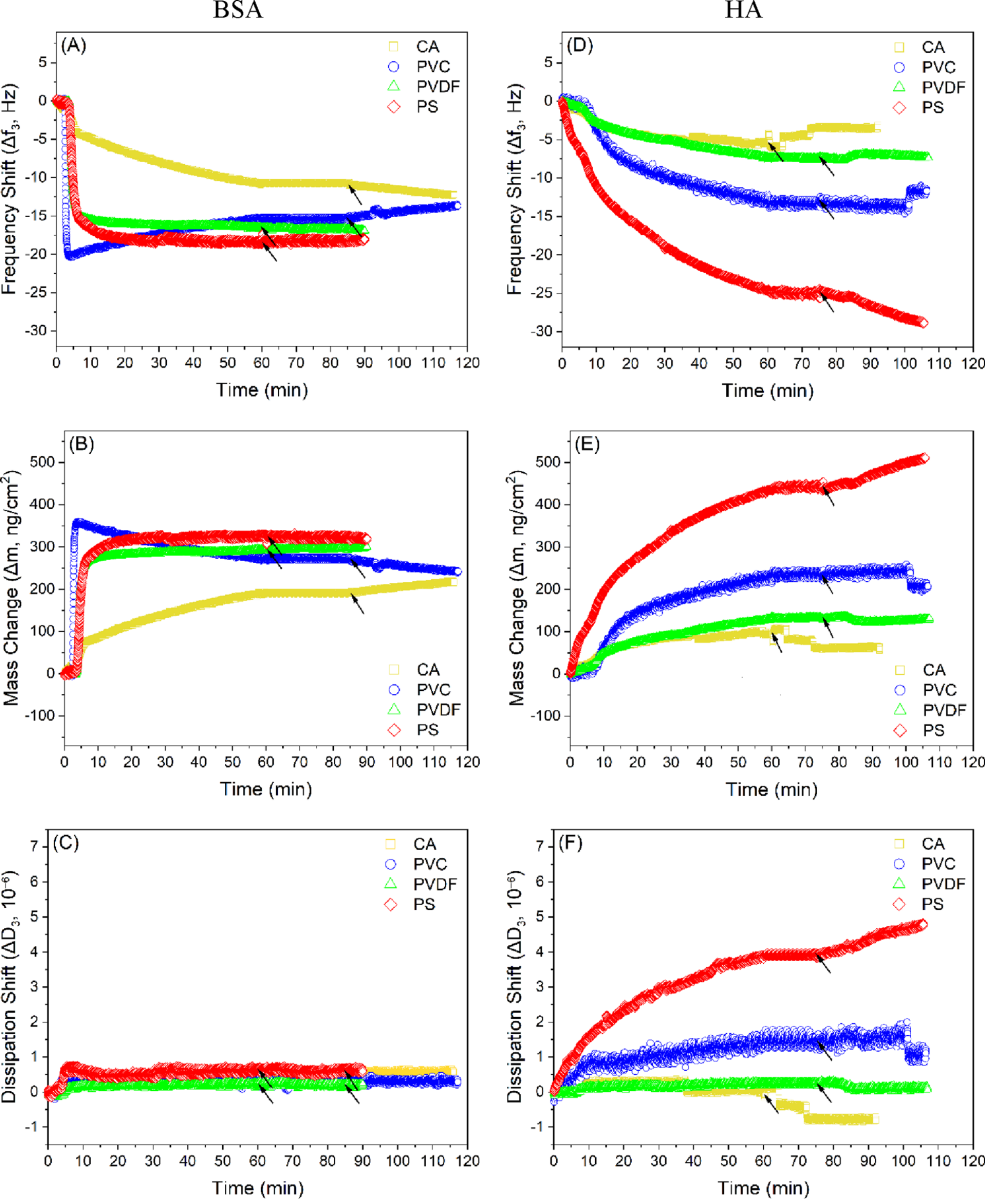
Frequency, mass, and dissipation shift curves for the adsorption of (A–C) BSA and (D–F) HA aqueous solutions onto the polymer films. At time of 60 min, DI water was injected into the system for rinsing. Arrows in the figures show the time at which rinsing was started. CA: cellulose acetate, PVC: polyvinyl chloride, PVDF: polyvinylidene fluoride, and PS: polysulfone.

The total mass of BSA adsorbed onto the polymer films in the QCM-D measurements, at equilibrium, was obtained in the order of PS > PVDF > PVC > CA. The mass shift (Δ*m*) quantified for PS was 41, 17, and 10% higher than the mass shifts obtained for CA, PVC, and PVDF, respectively (Table 6 and Figure 7). A similar trend was observed between the BSA-normalized adhesion forces (*F*_AFM_/*R*) and the QCM-D mass shifts (Δ*m*). Similarly, the same trend (*R*^2^ = 0.69) was observed between the BSA-normalized adhesion energies (*W*_AFM_/*R*) and the QCM-D mass shifts (Δ*m*). When the QCM-D mass shifts (Δ*m*) of BSA were plotted versus each XDLVO component force (*F*^El^, *F*^LW^, and *F*^AB^) calculated at the minimum equilibrium distance of 0.157 nm, *R*^2^ values were found to be 0.05, 0.003, and 0.997, respectively (Figure S8). This indicates the dominance of the AB interactions and the better suitability of the XDLVO theory in describing and predicting the adsorption behavior of BSA onto the polymer films compared to the DLVO model. In addition, the rate of BSA adsorption onto the polymer films in the QCM-D measurements was found in the order of PVC > PVDF > PS > CA (Table 6 and Figure 7). The BSA adsorption rate measured for PVC was 98, 47, and 29% higher than the rates quantified for CA, PS, and PVDF, respectively. However, no trend was obtained between the adsorption rate of BSA and the energy barriers predicted by XDLVO. This is probably due to the γ_S_^–^ overestimation, as mentioned previously, which may result in a different ordering of the energy barriers.

**Table 6:** Frequency and Mass Shifts, Rate , and Change of Dissipation versus Frequency ( | Δ*D*_3_/(Δ*f*_3_/3) | ), for the Adsorption of 100 ppm BSA or HA Aqueous Solutions onto the Polymer Films

polymer film	biofoulant solution	Δ*f*_3_/3 (Hz)	Δ*m* (ng/cm^2^)	(Hz/min)	(ng/cm^2^·min)	|Δ*D*_3_/(Δ*f*_3_/3)| (10^–6^/Hz)
CA	BSA	–10.85	192.10	–0.47	8.27	0.0493
	HA	–5.450	96.46	–0.75	13.25	0.0563
PVC	BSA	–15.36	271.81	–18.96	335.57	0.0205
	HA	–13.02	230.45	–0.76	13.43	0.0802
PVDF	BSA	–16.69	295.35	–13.38	236.76	0.017
	HA	–7.31	129.45	–0.69	12.30	0.0342
PS	BSA	–18.46	326.68	–10.10	178.70	0.0262
	HA	–24.61	435.66	–1.82	32.27	0.1613

For HA, the total mass adsorbed onto the polymer films in the QCM-D measurements, at equilibrium, was found to be in the following order: PS > PVC > PVDF > CA. The mass shift (Δ*m*) quantified for PS was 78, 70, and 47% higher than the mass shifts obtained for CA, PVDF, and PVC, respectively (Table 6 and Figure 7). When excluding PVDF, perhaps due to its rougher surface, a similar trend (*R*^2^ = 0.91) was observed between the HA-normalized adhesion forces (*F*_AFM_/*R*) and the QCM-D mass shifts (Δ*m*). When the QCM-D mass shifts (Δ*m*) of HA were plotted against each XDLVO component force (*F*^El^, *F*^LW^, and *F*^AB^) calculated at the minimum equilibrium distance of 0.157 nm, *R*^2^ values were found to be 0.001, 0.62, and 0.95, respectively (Figure S9). This indicates that the XDLVO theory can better fit the adsorption behavior of HA onto the polymer films than the DLVO model. Furthermore, the rate of HA adsorption onto the polymer films in the QCM-D measurements was found to be in the order of PS > PVC > CA > PVDF (Table 6 and Figure 7). The HA adsorption rate measured for PS was 59, 58, and 62% higher than the rates quantified for CA, PVC, and PVDF, respectively. The highest rate of PS was consistent with the lowest XDLVO energy barrier among the polymer films. The other three polymer films (CA, PVC, and PVDF) had comparable rates despite the notable differences seen in their energy barriers.

**Figure 8:**
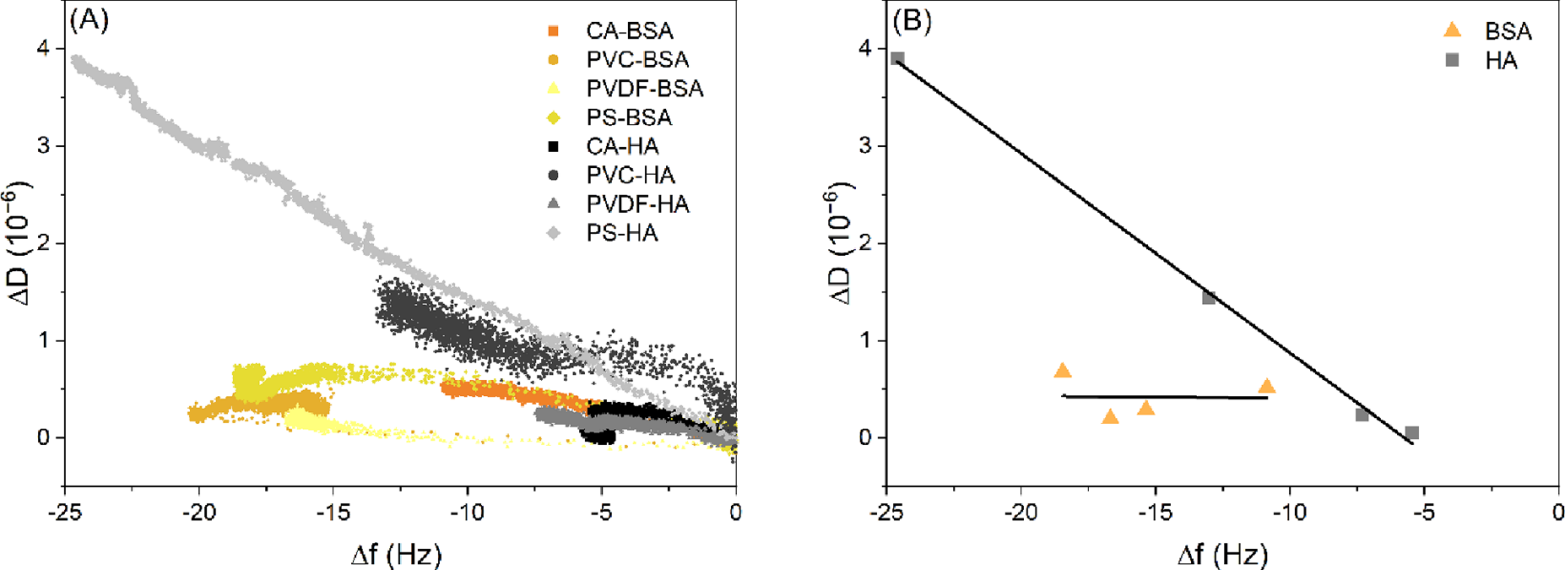
Change of dissipation versus frequency, |Δ*D*/Δ*f*|: (A) during the adsorption process of BSA and HA onto the polymer films and (B) at adsorption equilibrium. The steeper slopes of HA indicate a softer layer with an open structure. CA: cellulose acetate, PVC: polyvinyl chloride, PVDF: polyvinylidene fluoride, and PS: polysulfone.

The mass shifts (Δ*m*) quantified for BSA were higher than those obtained for HA, except for PS (Table 6 and Figure 7). In addition, the adsorption rates measured for BSA were higher than those for HA, except for CA. This can also be reflected by the higher dissipation shifts (Δ*D*) observed for HA in comparison to BSA (Figure 7C,F). The higher Δ*D* values point out the viscoelastic nature of the HA adsorbed layer compared to the BSA layer. This was also confirmed by plotting the change of dissipation versus frequency ( | Δ*D*_3_/(Δ*f*_3_/3) | ) during the adsorption process (Figure 8A) and at equilibrium or saturation (Figure 8B). The steeper slopes for HA in Figure 8 demonstrated that HA adsorbed in the form of a soft layer with an open structure. This can be attributed to the more negative charge and the higher γ_S_^–^ (higher hydrophilic repulsion with the polymer films) of HA molecules compared to BSA molecules (Table 1). This gives rise to higher energy barriers for HA than BSA, prohibiting the close proximity of HA molecules and the deposition of less compact structures on the polymer films as compared with the BSA.

Furthermore, in Figure 7D, the adsorbed HA molecules were desorbed during the rinsing step, as seen from the subsequent drops in the frequency shifts, indicating the weakness of HA structures compared to the BSA-adsorbed layers. However, such drops in the frequency shifts were less detected in the desorption or rinsing step of BSA. Proteins, such as BSA, usually undergo a two-step process in protein–surface interactions. Initially, the hydrophobic surface triggers the unfolding of the protein to a more open and less structured state. Afterward, the expanded structure increases the affinity sites between the protein and the surface. Thus, it exposes the hydrophobic core groups facilitating the adsorption and aggravating the aggregation of proteins on the surface.^68,69^ Such conformational or structural changes in proteins can explain the denser and more irreversible layers formed on the polymer films.

**Figure 9:**
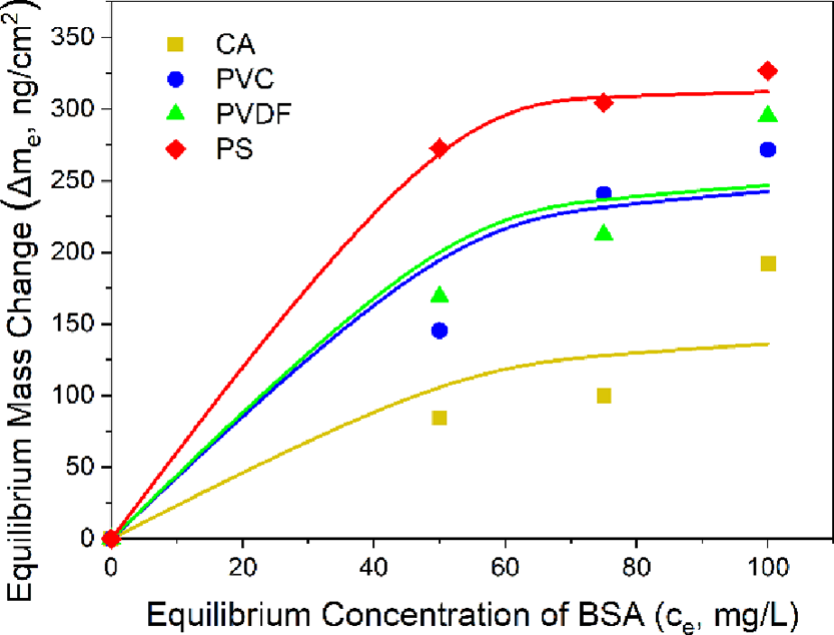
Adsorption equilibrium isotherms of BSA onto the four polymer films at a temperature of 294 K, no pH adjustment, and an adsorption time of 60 min. The solid lines represent the Langmuir model fits (eq 33). CA: cellulose acetate, PVC: polyvinyl chloride, PVDF: polyvinylidene fluoride, and PS: polysulfone.

The Langmuir isotherm fits are shown in Figure 9, and the corresponding fitting parameters are listed in Table 7. The polymer PS showed the highest maximum adsorption capacity (Δ*m*_max_) and the highest Langmuir constant (*k*_L_) compared to the other polymers. In comparison, CA showed the lowest Δ*m*_max_ and *k*_L_. The Δ*m*_max_ of PS was 49, 14, and 14% higher than that of CA, PVC, and PVDF, respectively. Similarly, the *k*_L_ of PS was 83, 77, and 73% higher than that of CA, PVC, and PVDF, respectively. The trend observed in Δ*m*_max_ and *k*_L_ agrees with those observed in the AFM adhesion forces and energies and the QCM-D adsorption amounts. The adsorption equilibrium data was well-fitted with the Langmuir isotherm model for most polymers, indicating that a monolayer adsorption of BSA occurred on the polymer films. These results agree with a previous study by Hashino et al., where it was found that the higher hydrophilicity of polymer films results in a decrease in the amount of BSA adsorbed onto the polymer film.^70^

**Table 7:** Fitting Parameters for the Langmuir Isotherm of BSA Adsorption onto the Polymer Films

parameter	CA	PVC	PVDF	PS
Δ*m*_max_ (ng/cm^2^)	163.93	277.78	277.78	322.58
*k*_L_ (L/mg)	0.0488	0.0688	0.0795	0.295
*R*^2^	0.63	0.81	0.85	0.95

In addition, the adsorption equilibrium constants (*K*_eq_) and the standard free energy changes of BSA adsorption onto the polymer films (Δ*G*_ads_^°^) are summarized in Table 8. The polymer PS showed the highest *K*_eq_ and the highest Δ*G*_ads_^°^ compared to the other polymers. In comparison, CA showed the lowest *K*_eq_ and Δ*G*_ads_^°^. The *K*_eq_ of PS was 83, 77, and 73% higher than that of CA, PVC, and PVDF, respectively. Similarly, the Δ*G*_ads_^°^ of PS was 9, 7, and 6% higher than that of CA, PVC, and PVDF, respectively. All *K*_eq_ values were >1 and all Δ*G*_ads_^°^ < 0, which demonstrated that the adsorption process of BSA onto the polymer films was favored. The trend found for *K*_eq_ and Δ*G*_ads_^°^ agrees with those found for the AFM adhesion forces and energies and the QCM-D adsorption amounts.

**Table 8:** Summary of the Estimated Adhesion Energy Parameters between the Polymer Films and the Model Biofoulant BSA in Water^a^

	polymer film			
parameter	CA	PVC	PVDF	PS
Δ*G*_132_^adh^ (mJ/m^2^)	11.68	–0.34	–0.36	–7.46
*a*_0_ (nm)^b^	–75.76	21.10	21.71	61.09
*W*^adh^ (aJ)	0	–0.47	–0.53	–87.45
*W*_AFM_ (aJ)	–11.94	–14.85	–25.08	–45.34
*W*_AFM_/*R* (pJ/m)	–4.776	–5.941	–10.031	–18.135
no. of biofoulant molecules	0	36.4	38.5	304.6
*K*_eq_ (dimensionless)	1.80 × 10^8^	2.54 × 10^8^	2.93 × 10^8^	1.09 × 10^9^
Δ*G*_ads_^°^ (J/mol)	–46,485.31	–47,326.49	–47,677.89	–50,887.45
Δ*G*_ads_^°^ (aJ)	0	–2.86	–3.05	–25.74

a The measurements were performed in DI water with an average pH value of 5.6.

b The negative sign of the contact radius ()of CA resulted from a repulsive value.

### A Correlation between AFM Microscopic Adhesion Energy and Macroscopic Adhesion Energy between BSA and Polymer Films in Water

**Figure 10:**
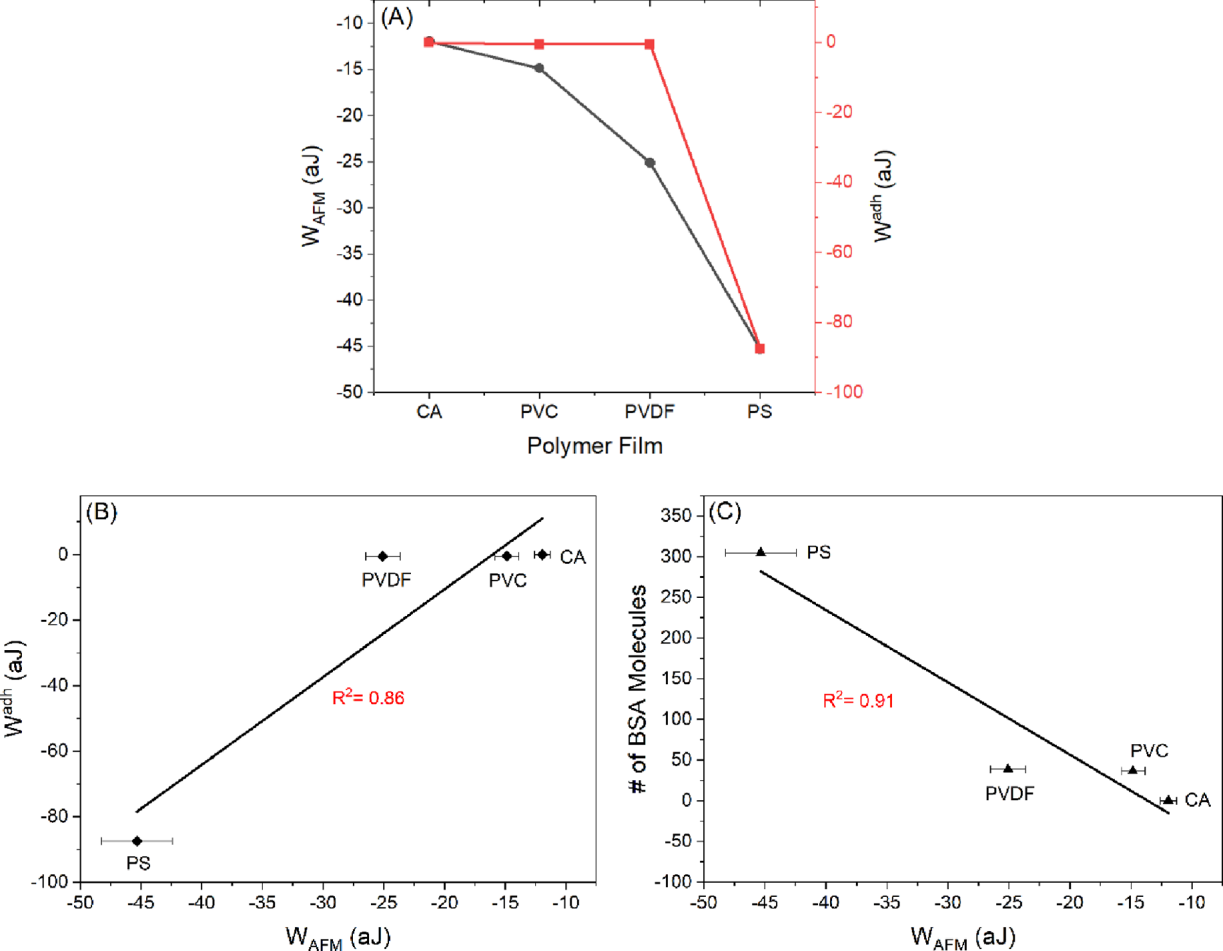
(A) Relationship between the mean of the AFM adhesion energies (*W*_AFM_) quantified between BSA-functionalized colloidal probes and the polymer films in water and the macroscale adhesion energies calculated from the contact angle measurements using a thermodynamic-based approach (*W*^adh^), showing a similar trend between the two quantities. (B) Scatter plot showing a linear correlation between *W*_AFM_ and *W*^adh^: (*W*^adh^ (aJ) = 2.6787 *W*_AFM_ (aJ) + 42.985, *R*^2^ = 0.86). (C) Scatter plot showing a linear correlation between *W*_AFM_ and number of BSA molecules: (no. of BSA molecules = −8.8936 – 121.27, *R*^2^ = 0.91). Errors reported in the figures are the standard errors of the mean. CA: cellulose acetate, PVC: polyvinyl chloride, PVDF: polyvinylidene fluoride, and PS: polysulfone.

A similar trend (Figure 10A) was observed between the BSA macroscopic adhesion energies (*W*^adh^) estimated using the macroscopic contact angle measurements (eq 27, Table 8) for the four polymer films, which were then multiplied by the circular contact area obtained by using the JKR model (eq 29, Table 8) and the BSA adhesion energies (*W*_AFM_) obtained from the AFM retraction curves (eq 1, Table 8). When the two quantities were plotted against each other, a linear correlation (*R*^2^ = 0.86) was obtained (Figure 10B). However, when the Hertz model was applied to calculate the circular area of contact, a weaker correlation (*R*^2^ = 0.65) was acquired between *W*_AFM_ and *W*^adh^ (data not shown). This demonstrates the suitability of the JKR model for systems that apply large-areal colloidal probes. This also reveals the essential role that the surface energy plays in the adhesion contact area and, thus, in the adhesion magnitude, and which exceeds the role played by the stiffness of the polymer film sample.

Additionally, the adhesion energies (*W*_AFM_) quantified for BSA were found to correlate well with the number of BSA molecules available in the contact region (*R*^2^ = 0.91). In contrast, the adhesion forces (*F*_AFM_) or the normalized adhesion forces (*F*_AFM_/*R*) quantified for BSA had a weaker correlation (*R*^2^ = 0.52) with the number of BSA molecules. This is probably because the AFM adhesion energy is required to unfold and break all the bonds (all adhesion events) and peel the probe away from the surface. In contrast, the adhesion force is recorded in a single adhesion event (the maximum attractive force in our case).

### A Correlation between Normalized AFM Adhesion Energy and Free Energy of Adsorption of BSA onto Polymer Films in Water

The standard Gibbs free energy change of adsorption (Δ*G*_ads_^°^) is the change in the free energy or chemical potential of the adsorbate as it transfers from the solution state under standard conditions (i.e., 1 atm of pressure, the liquid or gas to be pure, and the solution to be at 1 M concentration) to the adsorbed state under standard conditions. The amount of the standard free energy of adsorption reflects how far the system is at its standard conditions from equilibrium. The more negative the value of Δ*G*_ads_^°^, the farther the system is from equilibrium. Therefore, it can provide information on the extent or limit of adsorptive fouling. Higher negative values of Δ*G*_ads_^°^ can indicate higher biofouling potentials of the biofoulant toward the surface being investigated.

**Figure 11:**
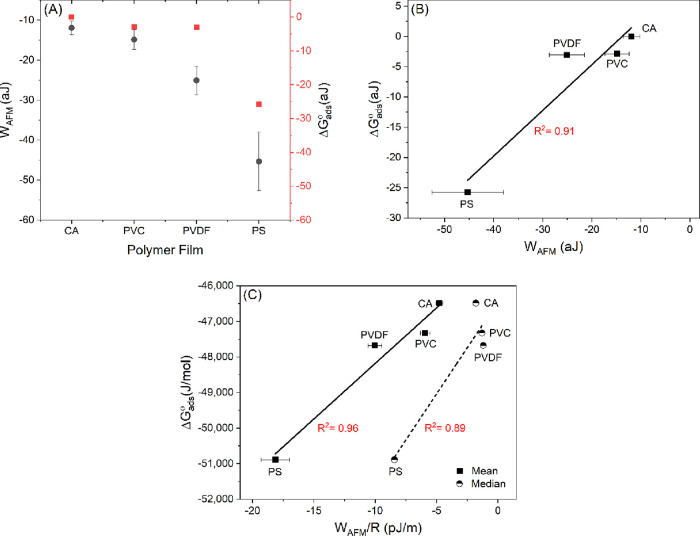
(A) Relationship between the mean of the AFM adhesion energies (*W*_AFM_) quantified between BSA-functionalized colloidal probes and the polymer films in water and the standard Gibbs free energies of adsorption (Δ*G*_ads_^°^), showing a similar trend between the two quantities. (B) Scatter plot showing a linear correlation between the mean of *W*_AFM_ versus Δ*G*_ads_^°^. The solid line is the linear regression fit of the mean of *W*_AFM_ versus Δ*G*_ads_^°^, represented by Δ*G*_ads_^°^ (aJ) = 0.7542 *W*_AFM_ (aJ) + 10.416, *R*^2^ = 0.91). (C) Scatter plot showing a linear correlation between both the mean and median of *W*_AFM_/*R* versus Δ*G*_ads_^°^. The solid line is the linear regression fit of the mean of *W*_AFM_/*R* versus Δ*G*_ads_^°^, represented by Δ*G*_ads_^°^ (J/mol) = 312.24 *W*_AFM_/*R* (pJ/m) – 45,059, *R*^2^ = 0.96). Errors reported in the figures are the standard errors of the mean. The dashed line is the linear regression fit of the median of *W*_AFM_/*R* versus Δ*G*_ads_^°^, represented by Δ*G*_ads_^°^ (J/mol) = 1294.5 *W*_AFM_/*R* (pJ/m) – 46,447, *R*^2^ = 0.89). CA: cellulose acetate, PVC: polyvinyl chloride, PVDF: polyvinylidene fluoride, and PS: polysulfone.

On the other hand, the AFM adhesion energy represents the energy required to transfer the BSA from the adsorbed state to the stretched state. Since the exact number of BSA molecules involved during the detachment of the colloidal probe away from the polymer sample surface is not known, and only the maximum number of molecules can be predicted based on the approximated contact area between the probe and the sample, it is not possible to estimate the free energy change of adsorption per mole of BSA molecules directly from the area under the velocity-independent force plateau, such as the case in single-molecule AFM measurements.^71,72^ However, to compare between the BSA standard free energy changes of adsorption and the AFM adhesion energies, and to estimate the thermodynamic driving force to reach equilibrium in the AFM experiments, assuming that the stretched state and the free bulk solution state are close to each other and that hysteresis is negligible, the BSA standard free energies of adsorption (Δ*G*_ads_^°^) obtained using equilibrium adsorption studies for the four polymer films was multiplied by the maximum predicted number of BSA moles in the contact area in AFM studies. A qualitative agreement (Figure 11A) was observed between the BSA standard free energy changes (Δ*G*_ads_^°^) in units of aJ and the AFM adhesion energies (W_AFM_) of BSA (Table 8). A linear correlation (*R*^2^ = 0.91) was shown with the mean of *W*_AFM_ (Figure 11B).

Interestingly, when the BSA standard free energy changes of adsorption (ΔG_ads_^°^) in (J/mol) and the AFM normalized adhesion energies (W_AFM_/R) of BSA were plotted against each other, a linear correlation (R^2^ = 0.96) was obtained with the mean and median of W_AFM_/R (Figure 11C). This correlation can be applied to predict the standard free energy changes of BSA adsorption and hence the equilibrium constants of adsorption from the normalized adhesion energies measured by the colloidal probe technique. However, more data points need to be added in the future to improve the equation’s predictive potential. A similar correlation has been reported in the literature for peptide–surface interactions between the adsorption free energy change measured by surface plasmon resonance spectroscopy and the adhesion (desorption) forces measured by AFM using peptide-functioned sharp tips.^72,73^ The authors pointed out that since the same standardized methodology was used to functionalize the probes, similar probe tip densities of tethered peptides, although unknown, can be expected and thus can be correlated with the free energy of adsorption. In our study, all the AFM colloidal probes were coated using the same procedure. Hence, a similar coating density of BSA for all the colloidal probes will be expected and can be related to the free energy of adsorption.

### Estimation of Hamaker Constant and DLVO/XDLVO Analysis Using Hansen Solubility Parameters

The porosity of solid materials sometimes prevents the direct measurement of their contact angles. Therefore, we propose an indirect approach to approximate the components of the surface energies required for Hamaker constant calculation and the DLVO/XDLVO analyses from Hansen dissolution data. Such an indirect approach would be of interest when measuring the surface energies of biopolymers or biofoulants, which are characterized by high porosities, and which is challenging to prepare nonporous films from them for surface contact angle measurements. The conventional XDLVO analysis is based on quantifying surface energies using a minimum of three solvents. However, a numerical linear fitting is needed to obtain the best solution when more than three liquids are used. Furthermore, it has been speculated that the three acquired components of surface energies depend on the number and choice of these liquids.^74,75^ In addition, the negative values sometimes obtained for the square roots of the acid–base parameters are problematic.^74^ Therefore, dissolution tests using a range of solvents would be a more straightforward analysis.

**Figure 12:**
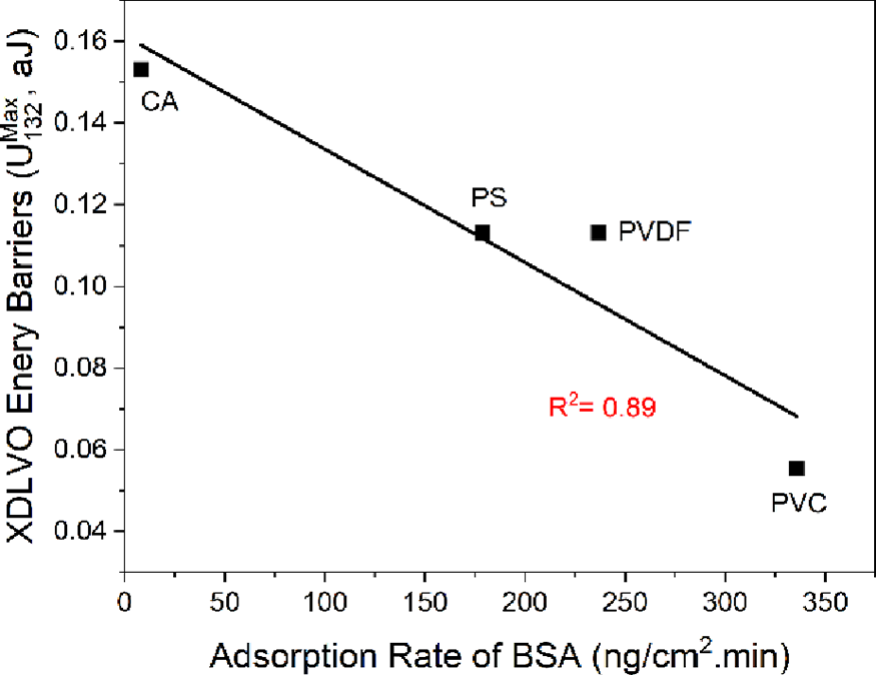
Scatter plot showing a linear correlation between the adsorption rate of BSA determined from QCM-D and the XDLVO energy barriers calculated between BSA and the four polymer films in water (*U*_132_^max^ (aJ) = −0.0003  (ng/cm^2^·min) + 0.1614, *R*^2^ = 0.89). CA: cellulose acetate, PVC: polyvinyl chloride, PVDF: polyvinylidene fluoride, and PS: polysulfone.

When eqs 8 and 9 were used to estimate the dispersive component of the surface energy from the dispersive Hansen solubility parameter, eq 9 predicted a closer value of γ^LW^ to that of water (21.8 mJ/m^2^) (Table 9). Therefore, we based the rest of our calculations on the values obtained from eq 9. As seen in Table 9, the calculated values of Δ*G*_132_^adh^ are all negatively signed, indicating a favorable and spontaneous adhesion of BSA to the polymer films. This is consistent with what was inferred from the AFM adhesion events and the QCM-D frequency shifts. When the XDLVO analysis was performed using the Hamaker constants and the AB components of the free energies (Δ*G*_132_^AB^) calculated from the dissolution data, a better correlation (*R*^2^ = 0.86) was observed between the QCM-D adsorption rate of BSA and the energy barriers predicted by the XDLVO model (Figure 12). Both PVDF and PS had close adsorption rates and energy barriers. It should be noted that an approximative equation, such as eq 11, will make the prediction less precise and raise the error in the estimated parameters. Thus, empirical equations connecting the acidic (γ^+^) and basic (γ^–^) components of the surface energies with Hansen solubility parameters will be of critical and practical importance and can improve the accuracy of the predictive analysis method.

**Table 9:** Summary of the Estimated Interfacial Surface Energies, the Free Energy of Adhesion per Unit Area (Δ*G*_132_^adh^), and the Predicted XDLVO Energy Barriers between the Polymer Films and the Model Biofoulant of BSA in Water^a^

	polymer film				biofoulant	
parameter	CA	PVC	PVDF	PS	BSA	DI water
γ^LW^ (mJ/m^2^) (eq 8)	26.36	37.79	25.61	39.25	132.93	17.51
γ^LW^ (mJ/m^2^) (eq 9)	25.42	29.52	25.07	29.91	38.32	20.09
γ^AB^ (mJ/m^2^) (eq 12)	8.80	6.87	8.81	5.47	15.20	26.30
Δ*G*_132_^AB^ (mJ/m^2^) (eq 11)	–5.31	–6.16	–5.31	–6.85		
Δ*G*_132_^LW^ (mJ/m^2^) (eq 14)	–1.91	–3.25	–1.79	–3.37		
Δ*G*_132_^adh^ (mJ/m^2^) (eq 15)	–7.23	–9.41	–7.10	–10.23		
*U*_132_^max^ (aJ)	0.1532	0.0554	0.1132	0.1131		

a The measurements were performed in DI water with an average pH value of 5.6.

## Conclusions

Model biofoulant-coated colloidal AFM probes were employed to investigate biofouling mechanisms for a set of commonly used polymer films in membrane synthesis: CA, PVC, PVDF, and PS. Two model biofoulants, i.e., BSA and HA, were selected. The mean values of the normalized adhesion forces quantified between BSA and the four polymer films were higher than the corresponding mean values quantified for HA. When the XDLVO analyses were applied to break down the overall adhesion interactions between the biofoulants and the polymer films into their component interactions, i.e., electrostatic, Lifshitz–van der Waals, and Lewis acid–base interactions, the adhesion of the two model biofoulants to the four polymer films was found to be governed by the Lewis acid–base interactions, which is the net of the attractive hydrogen bonding attractions and either the attractive hydrophobic attractions or the repulsive hydration interactions. The colloidal AFM adhesion measurements can be used as an assessment method to evaluate polymer film biofouling, as evidenced by its agreement with the actual adsorption behavior, which was investigated by QCM-D. The BSA adhesion strengths and adsorption quantities on the polymer films were ranked in the following order: PS > PVDF > PVC > CA. In comparison, the HA adhesion strengths were in the order of PVDF > PS > PVC > CA, and its adsorption quantities were in the order of PS > PVC > PVDF > CA. The model biofoulant BSA was found to adsorb onto the polymer films in more significant amounts, at higher rates, and in a denser form than HA. This was also confirmed by HA’s weaker and reversible adsorption, as it can be desorbed during the rinsing step. The XDLVO model was found to better describe and predict the AFM colloidal probe adhesion data and the adsorption behavior of BSA onto the polymer films than the DLVO model. The macroscopic adhesion energies (*W*^adh^) estimated from the macroscopic contact angle measurements and the AFM adhesion energies (*W*_AFM_) of BSA were linearly related (*R*^2^ = 0.86). Furthermore, the adhesion energies (*W*_AFM_) quantified for BSA were found to have a more linear relationship with the number of BSA molecules available in the contact region (*R*^2^ = 0.91) than the adhesion forces (*R*^2^ = 0.52). In addition, a linear correlation (*R*^2^ = 0.96) was attained for BSA between the standard free energy changes of adsorption (Δ*G*_ads_^°^) estimated from the equilibrium QCM-D adsorption experiments and the AFM-normalized adhesion energies (*W*_AFM_/R) calculated from the AFM colloidal probe force measurements. Finally, an indirect approach was proposed to measure the surface energies of biopolymers or biofoulants characterized by high porosities and which are challenging to prepare nonporous films from. A better linear correlation (*R*^2^ = 0.89) was obtained between the QCM-D adsorption rate of BSA and the energy barriers predicted by the XDLVO analysis performed based on Hansen dissolution data. However, empirical relations between individual AB surface energy components (γ^–^ and γ^+^) and Hansen solubility parameters are predicted to enhance the accuracy of the estimated results. Overall, these findings highlight the critical role of AB repulsion interactions, mainly the γ^–^ parameter, in controlling the biofouling of polymeric membranes.

## Supporting Information Available

Nine elements are available. (Figure S1) Fluorescence microscopy images of control uncoated amine silica spheres and FITC-labeled BSA coated probe after it was used in AFM force measurements; (Table S1) roughness parameters measured for the polymer films by atomic force microscopy (AFM); (Figure S2) representative AFM roughness images of the polymer films; (Table S2) surface tension component parameters of the liquids used in contact angle measurements; (Figure S3) examples of force–distance approach and retraction curves measured between a polymer film and a BSA-coated colloidal AFM probe in DI water and how the adhesion forces and the adhesion energies are measured; (Table S3) Hansen solubility parameters of the polymer films, the model biofoulant BSA, and DI water; (Figure S4) zeta potentials of uncoated amine-functionalized silica spheres, BSA/HA-coated silica spheres, and the polymer films as a function of solution pH; (Figure S5) shows AFM approach curves collected between the HA-coated colloidal probe and the CA film in DI water and in 0.5 M NaCl; (Figure S6) each XDLVO component force: electrostatic (El), Lifshitz–van der Waals (LW), and Lewis acid–base force versus the AFM-normalized adhesion force quantified between BSA-coated colloidal probes and the polymer films in water; (Figure S7) each XDLVO component force: electrostatic (El), Lifshitz–van der Waals (LW), and Lewis acid–base force versus the AFM-normalized adhesion force quantified between HA-coated colloidal probes and the polymer films in water; (Figure S8) each XDLVO component force: electrostatic (El), Lifshitz–van der Waals (LW), and Lewis acid–base force versus the QCM-D adsorbed masses of BSA onto the polymer films in water; (Figure S9) each XDLVO component force: electrostatic (El), Lifshitz–van der Waals (LW), and Lewis acid–base force versus the QCM-D adsorbed masses of HA onto the polymer films in water (PDF)
