# Supplementary material for: Interaction Mechanisms and Predictions of the Biofouling of Polymer Films: A Combined Atomic Force Microscopy and Quartz Crystal Microbalance with Dissipation Monitoring Study
Source: Langmuir. 2023 Apr 27;39(18):6592–612. doi: 10.1021/acs.langmuir.3c00587 (PMC10173465; doi:10.1021/acs.langmuir.3c00587)
Supplement: Supplementary file 1 — la3c00587_si_001.pdf [file la3c00587_si_001.pdf]

Interaction Mechanisms and Predictions of the Biofouling of Polymer Films: A Combined Atomic Force Microscopy and Quartz Crystal Microbalance with Dissipation Monitoring Study

Asma Eskhan[1], Neveen AlQasas[2], Daniel Johnson[3*]

[1]NYUAD Water Research Center, New York University Abu Dhabi (NYUAD), 129188 Abu Dhabi, UAE

[2]NYUAD Water Research Center, New York University Abu Dhabi (NYUAD), 129188 Abu Dhabi, UAE

[3]Civil Engineering Department, New York University Abu Dhabi, 129188 Abu Dhabi, UAE; NYUAD Water Research Center, New York University Abu Dhabi (NYUAD), 129188 Abu Dhabi, UAE

*corresponding author, Email: djj2026@nyu.edu

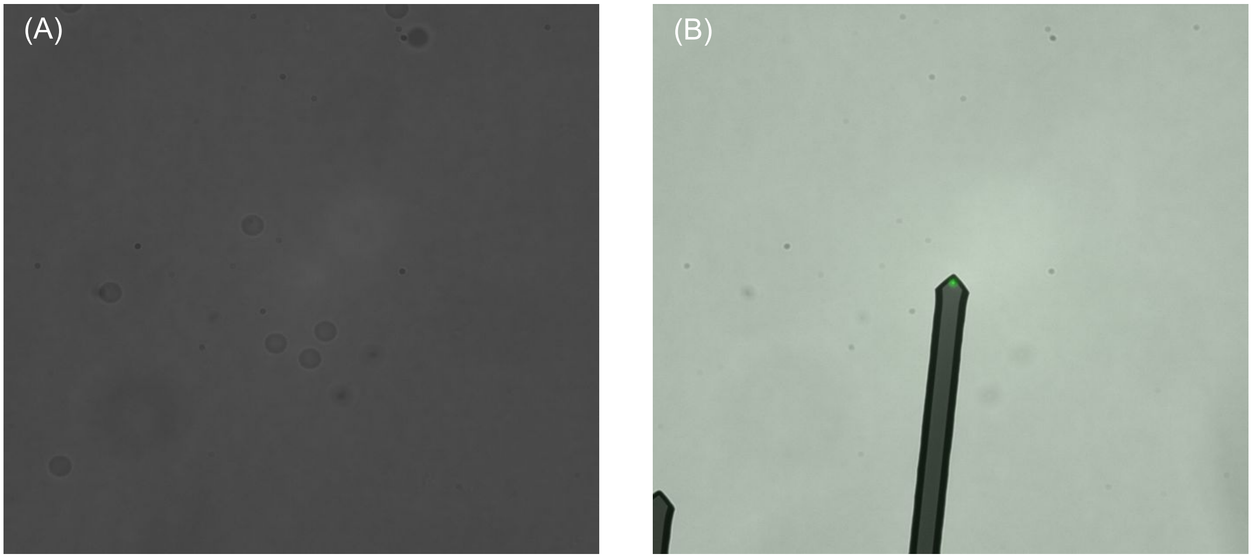

Figure S1. (A) Fluorescence microscopy image of amine-functionalized silica spheres before coating with BSA (control sample), where no fluorescence signal is shown. (B) Fluorescence microscopy image of a FITC-labeled BSA-coated colloidal AFM probe after it was used in AFM force measurements.

Table S1. Roughness parameters measured for the polymer films by AFM.[a]

Roughness parameter	CA	PVC	PVDF	PS
R_q (nm)[a]	37.93 ± 2.11	9.46 ± 2.90	246.00 ± 75.03	4.34 ± 1.13
R_a (nm)[a]	30.27 ± 1.02	7.56 ± 2.59	203.67 ± 67.10	2.34 ± 0.43
Roughness ratio = surface area/projected surface area[a]	1.03 ± 0.01	1.02 ± 0.01	1.08 ± 0.01	1.02 ± 0.01

[a] Errors reported represent the standard deviations of the means from triplicate measurements.

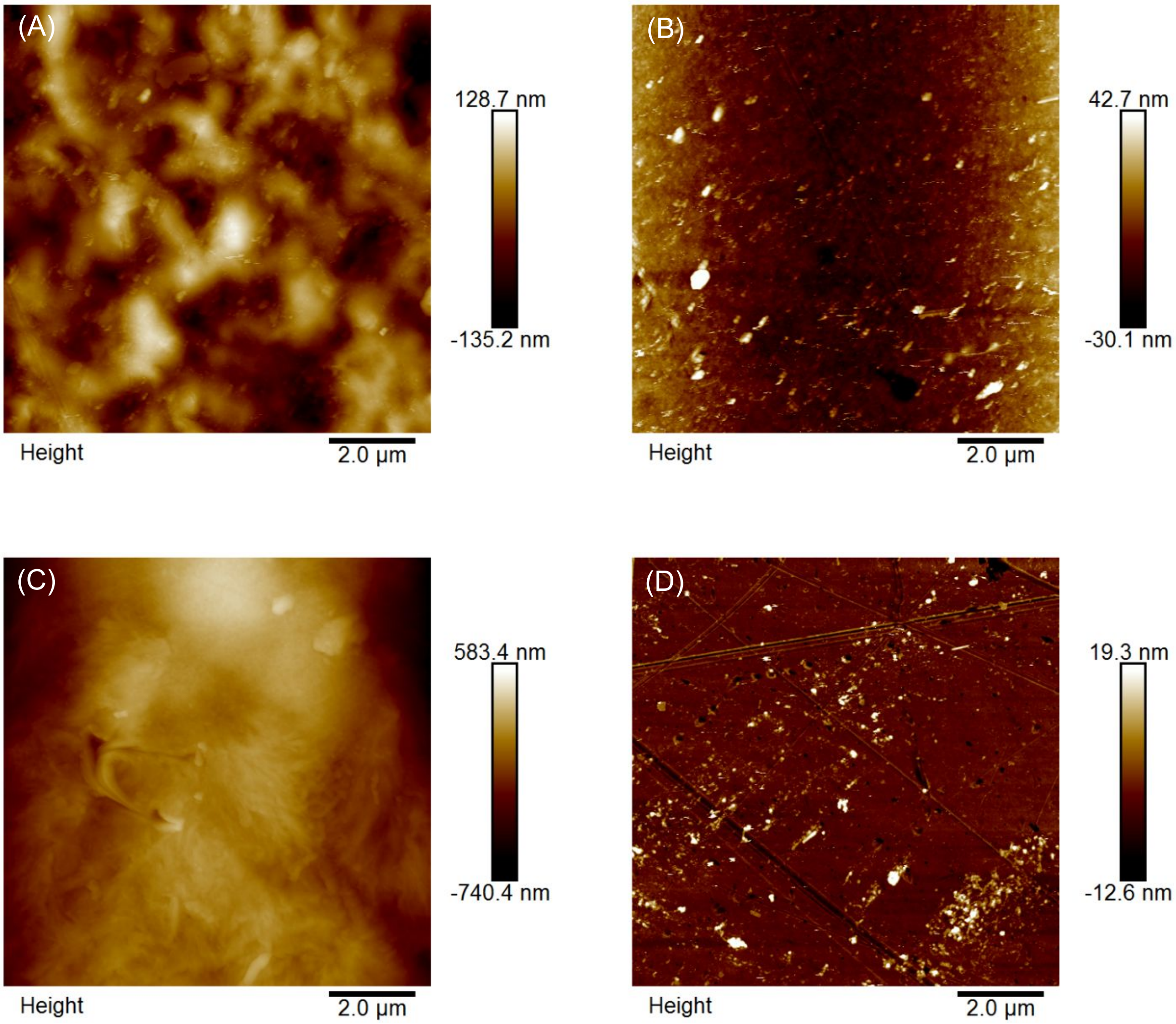

Figure S2. Representative AFM roughness images of the polymer films: (A): CA, (B) PVC, (C) PVDF, and (D) PS.

Table S2. Surface tension components (mJ/m^2) of the liquids used in contact angle measurements.

Liquid	γ_L^{TOT}	γ_L^{LW}	γ_L^{AB}	γ_L^{+}	γ_L^{-}
Water	72.8	21.8	51.0	25.5	25.5
Formamide	58	39	19	2.28	39.6
Diidomethane	50.8	50.8	0	0	0

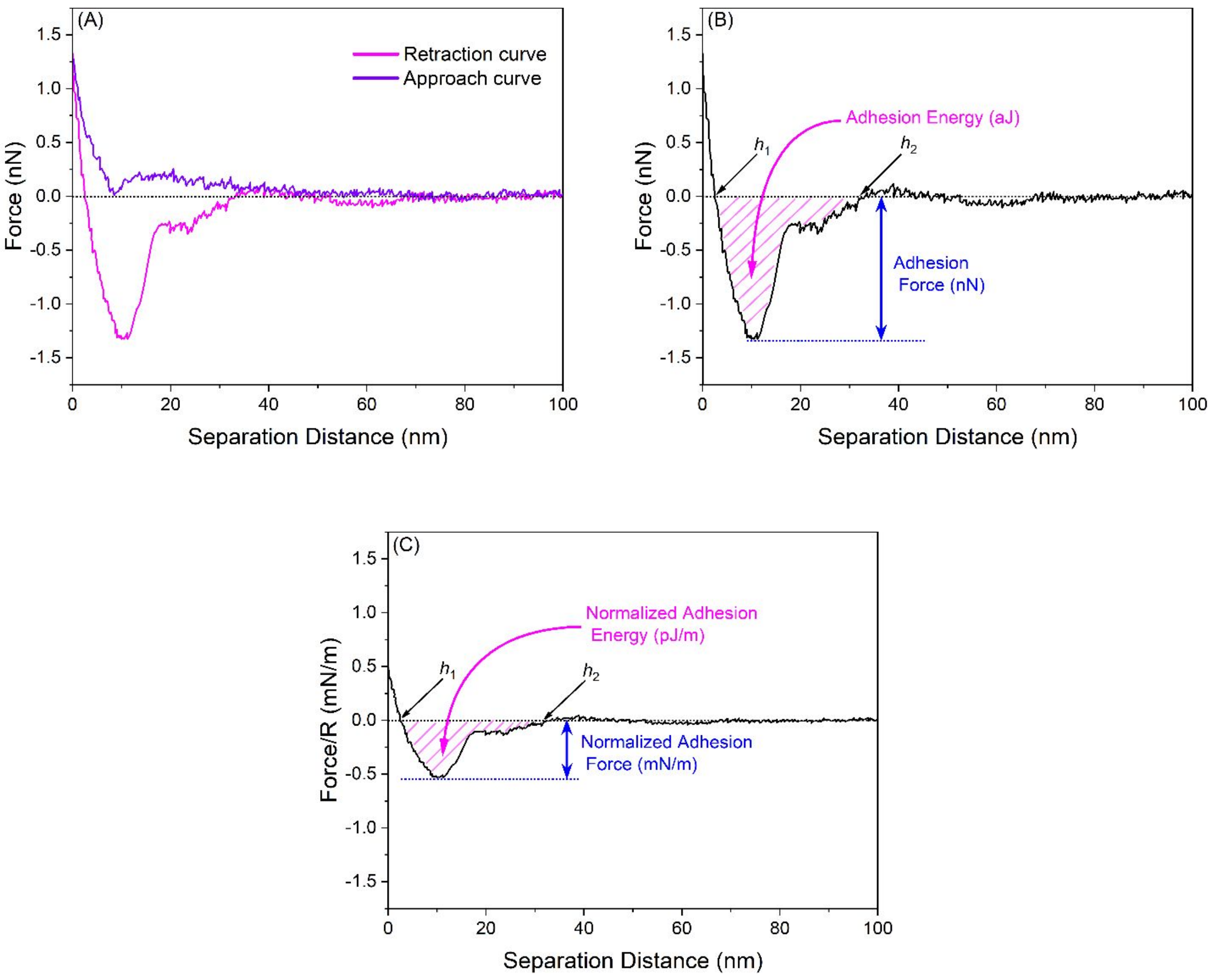

Figure S3. (A) Representative force–distance approach and retraction curves measured between a polymer film and a BSA-coated colloidal AFM probe in DI water. (B) A representative force–distance retraction curve measured between a polymer film and a BSA-coated colloidal AFM probe in DI water. The maximum rupture peak is the adhesion force and the shaded area is the adhesion energy calculated from Equation 2. (C) A representative normalized force–distance curve measured between a polymer film and a BSA-coated colloidal AFM probe in DI water. The maximum rupture peak is the normalized adhesion force and the shaded area is the normalized adhesion energy.

Table S3. Hansen solubility parameters of the polymer films, the model biofoulant BSA, and DI water.

Sample	δ_d (MPa)$^{0.5}$	δ_p (MPa)$^{0.5}$	δ_h (MPa)$^{0.5}$	δ_t (MPa)$^{0.5}$
CA	16.7	12.10	7.40	21.91
PVC	17.80	11.40	10.00	23.38
PVDF	16.60	9.80	9.90	21.67
PS	17.90	10.40	9.30	22.69
BSA	19.90	18.20	17.50	32.15
DI water	15.10	20.40	16.50	30.27

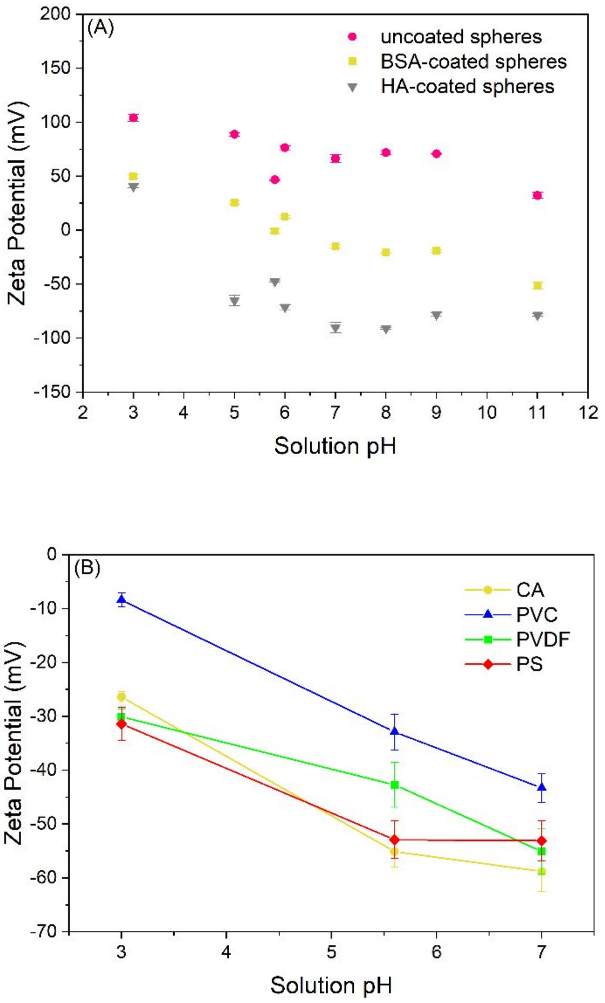

Figure S4. (A) Zeta potentials of uncoated and BSA/HA-coated amine-functionalized silica spheres as a function of solution pH. (B) Zeta potentials of the polymer films as a function of solution pH.

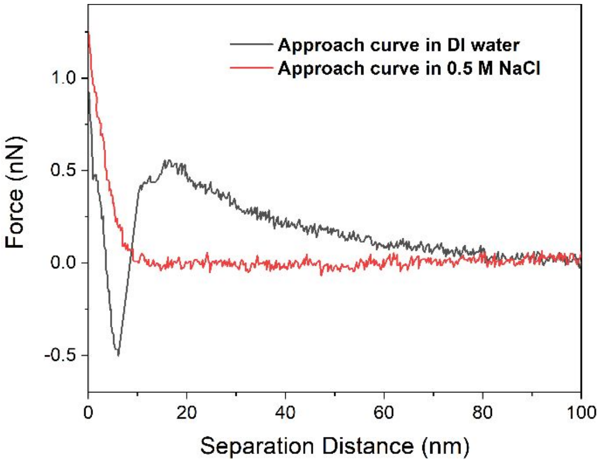

Figure S5. AFM Approach curves collected between HA-coated colloidal probe and CA film in DI water and in 0.5 M NaCl.

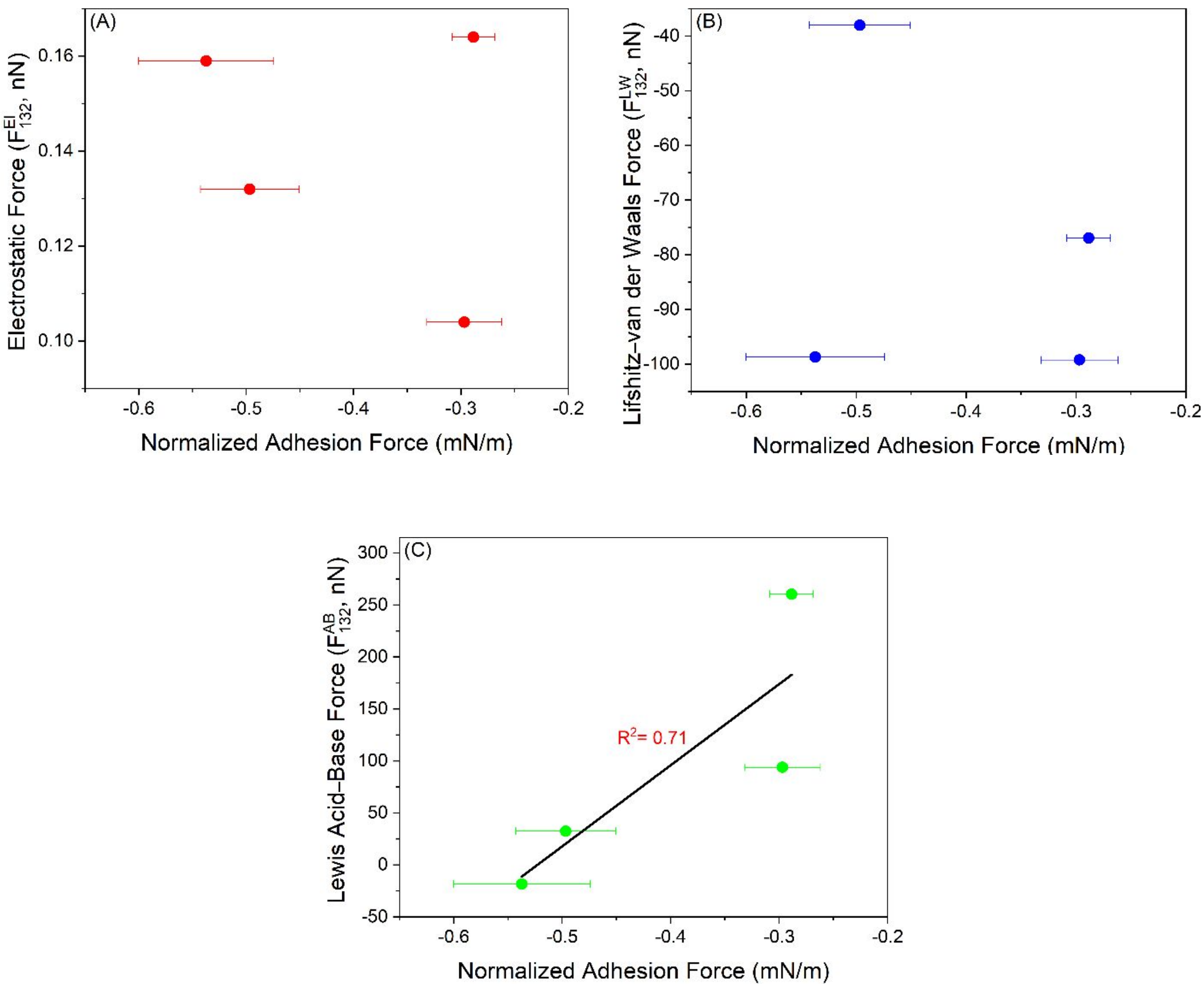

Figure S6. Scatter plots of each XDLVO component force: (A) Electrostatic (El), (B) Lifshitz–van der Waals (LW), and (C) Lewis acid–base force versus the AFM normalized adhesion force quantified between BSA-coated colloidal probes and the four polymer films in water. Errors reported in the figures are the standard error of the mean. R^2 values were found to be 0.07, 0.08, and 0.71 for El, LW and AB component plots, respectively.

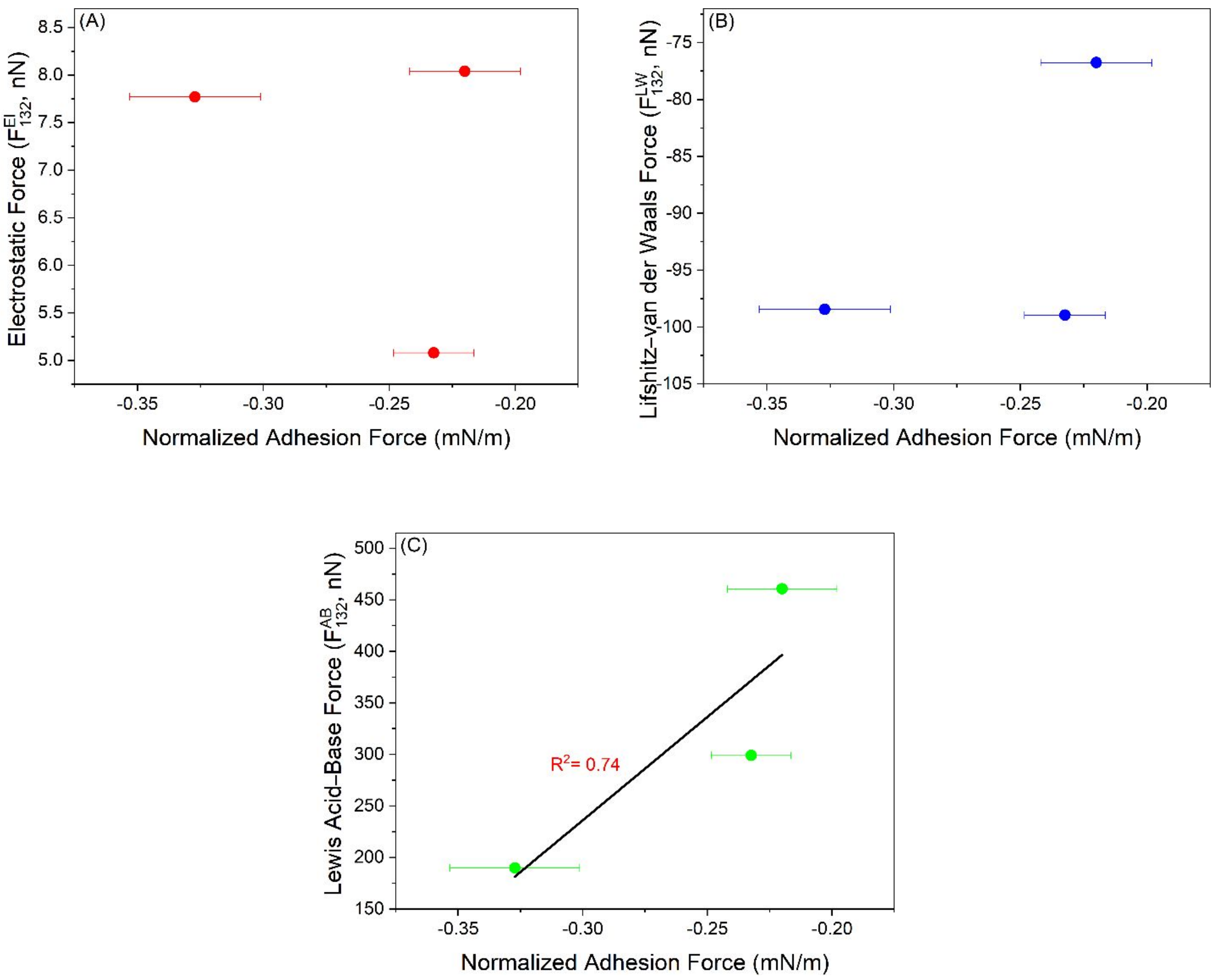

Figure S7. Scatter plots of each XDLVO component force: (A) Electrostatic (El), (B) Lifshitz–van der Waals (LW), and (C) Lewis acid–base force versus the AFM normalized adhesion force quantified between HA-coated colloidal probes and the polymer films, except PVDF, in water. Errors reported in the figures are the standard error of the mean. R^2 values were found to be 0.10, 0.33, and 0.74 for El, LW and AB component plots, respectively.

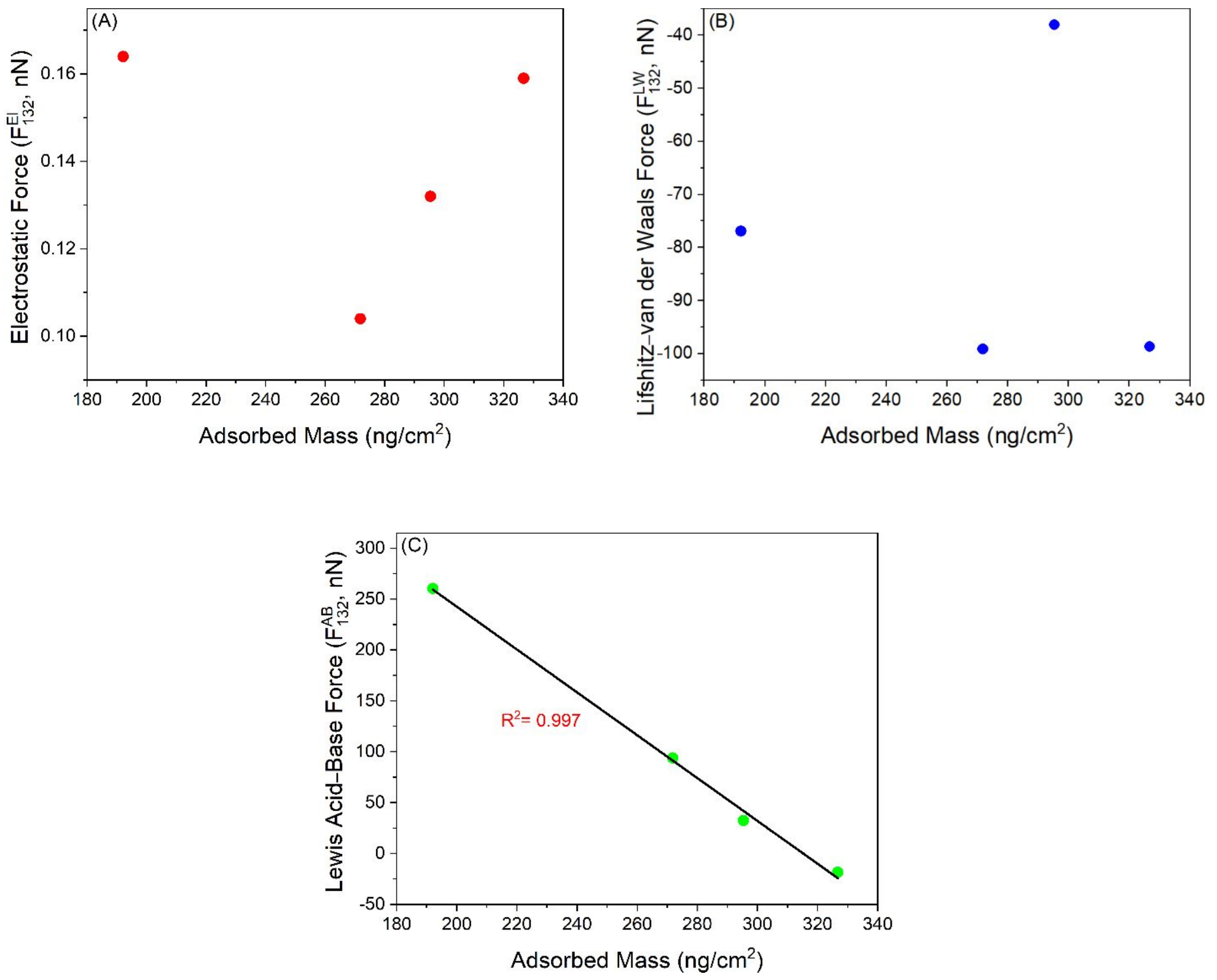

Figure S8. Scatter plots of each XDLVO component force: (A) Electrostatic (El), (B) Lifshitz–van der Waals (LW), and (C) Lewis acid–base force versus the QCM-D adsorbed masses of BSA onto the four polymer films in water. R^2 values were found to be 0.05, 0.003, and 0.997 for El, LW and AB component plots, respectively.

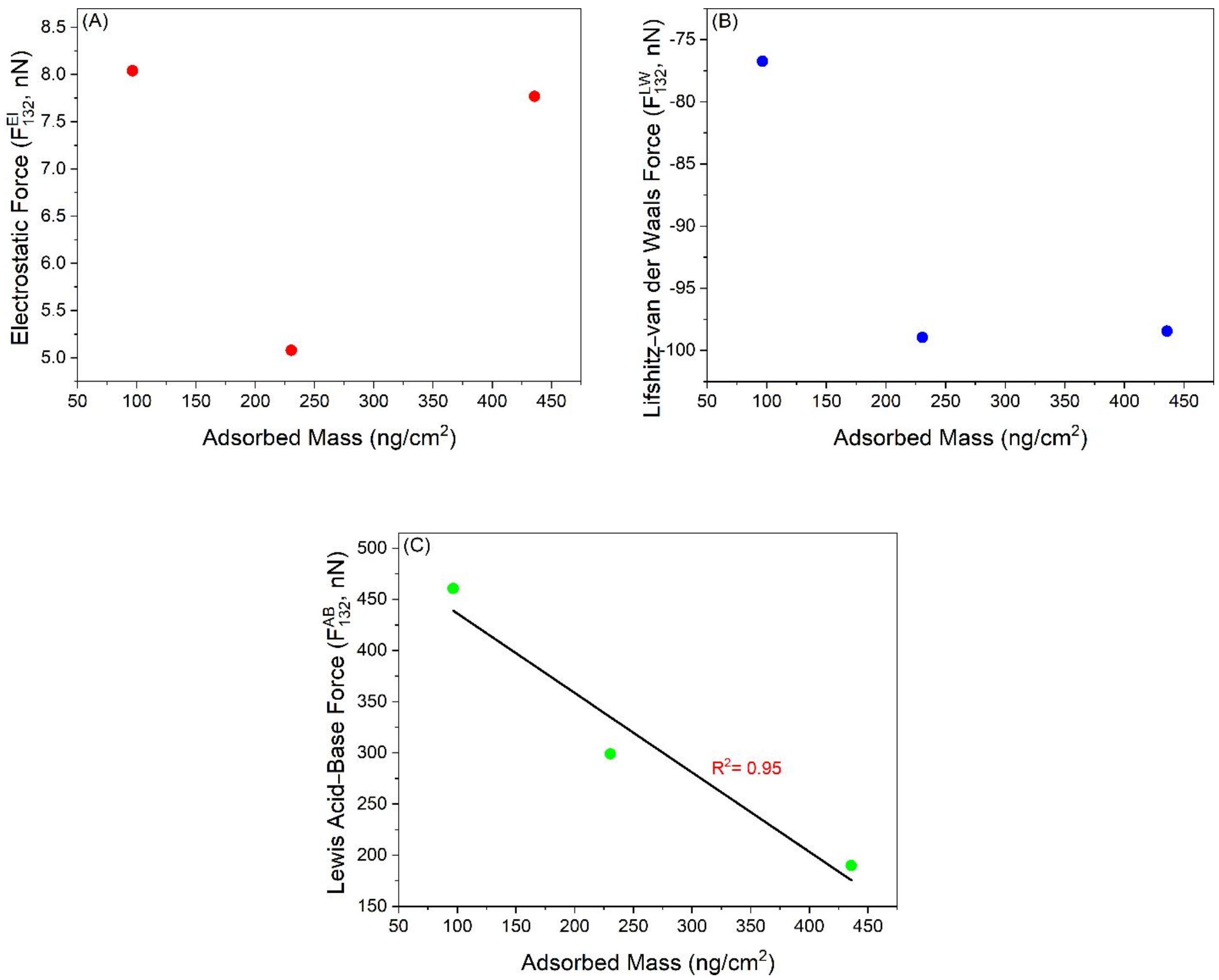

Figure S9. Scatter plots of each XDLVO component force: (A) Electrostatic (El), (B) Lifshitz–van der Waals (LW), and (C) Lewis acid–base force versus the QCM-D adsorbed masses of HA onto the polymer films, except PVDF, in water. R^2 values were found to be 0.001, 0.62, and 0.95 for El, LW and AB component plots, respectively.
